# Evolutionary conservation and diversification of auditory neural circuits that process courtship songs in *Drosophila*

**DOI:** 10.1038/s41598-022-27349-7

**Published:** 2023-01-07

**Authors:** Takuro S. Ohashi, Yuki Ishikawa, Takeshi Awasaki, Matthew P. Su, Yusuke Yoneyama, Nao Morimoto, Azusa Kamikouchi

**Affiliations:** 1 Graduate School of Science, Nagoya University, Nagoya, Aichi 464-8602 Japan; 2 School of Medicine, Kyorin University, Tokyo, 181-8611 Japan; 3 Institute for Advanced Research, Nagoya University, Nagoya, Aichi 464-8601 Japan; 4 Institute for Genetic Medicine, Hokkaido University, Sapporo, Hokkaido 060-0815 Japan; 5 Graduate School of Life Sciences, Tohoku University, Sendai, Miyagi 980-8577 Japan

**Keywords:** Auditory system, Sensory processing, Evolution, Animal behaviour, Neuroscience, Neural circuits

## Abstract

Acoustic communication signals diversify even on short evolutionary time scales. To understand how the auditory system underlying acoustic communication could evolve, we conducted a systematic comparison of the early stages of the auditory neural circuit involved in song information processing between closely-related fruit-fly species. Male *Drosophila melanogaster* and *D. simulans* produce different sound signals during mating rituals, known as courtship songs. Female flies from these species selectively increase their receptivity when they hear songs with conspecific temporal patterns. Here, we firstly confirmed interspecific differences in temporal pattern preferences; *D. simulans* preferred pulse songs with longer intervals than *D. melanogaster.* Primary and secondary song-relay neurons, JO neurons and AMMC-B1 neurons, shared similar morphology and neurotransmitters between species. The temporal pattern preferences of AMMC-B1 neurons were also relatively similar between species, with slight but significant differences in their band-pass properties. Although the shift direction of the response property matched that of the behavior, these differences are not large enough to explain behavioral differences in song preferences. This study enhances our understanding of the conservation and diversification of the architecture of the early-stage neural circuit which processes acoustic communication signals.

## Introduction

Many animal species utilize sound for communication. Acoustic signals used in courtship (i.e., courtship songs) are especially diversified during the evolutionary process even among closely related species^1^. Numerous playback experiments have shown that differences in courtship songs contribute to species recognition and mate choice (e.g. night monkey, greenish warbler, Darwin finch, and fruit fly^2–6^). Moreover, it has been suggested that song diversification accelerates speciation by preventing interspecific hybridization (e.g. cicada and Hawaiian cricket^7–9^ but see Chen and Wiens^10^). The divergence of acoustic communication thus plays a key role in animal diversification. Accordingly, signal-processing systems are expected to evolve to correspondingly recognize these diversified signals. However, how the auditory neural circuits that process courtship songs have evolved according to signal diversification has not yet been delineated.

Previous comparative studies in several animal species aiming to understand the neural mechanisms of sound processing evolution mostly focused on the responsible genetic loci or peripheral systems^11,12^. Comparative studies on auditory sensory cells of related species have revealed that the carrier frequency of these cells is tuned to the range of conspecific signals (e.g. fruit fly, mosquito, and anuran^11,13,14^). However, decoding more complex features, such as amplitude modulation or the sound intervals which characterize species specificity of courtship songs, is challenging when processing only with sensory cells. Indeed, the neural circuits downstream of sensory cells are known to play major roles in recognizing such complex acoustic features^15–18^. Thus, to understand how neural systems which process complex characteristics of conspecific songs have diversified, interspecific comparisons of the downstream neural circuit are required^19^.

*Drosophila melanogaster* is an excellent model for auditory studies due to its ability to discriminate sounds during acoustic communication and the availability of both genetic and connectomic tools for circuit dissection^20,21^. Males of most *Drosophila* species (including *D. melanogaster*) produce courtship songs by wing vibration^22–24^. Females, on the other hand, demonstrate an increase in copulation receptivity after hearing conspecific songs^6,25^, indicating the ability to discriminate sounds with conspecific characteristics.

The courtship songs of the *melanogaster* subspecies group (e.g., *D. melanogaster*, *D. simulans*, and *D. mauritiana*) generally comprise two components, trains of pulses (‘pulse songs’) and sequences of humming (‘sine songs’)^26^. Most of the behaviorally-relevant species-specific characteristics have been found in the pulse song^6^. Two major characteristics of the pulse song, inter-pulse interval (IPI; the intervals between two pulses) and intra-pulse frequency (IPF; a principal frequency component of the individual pulse), are different among the *melanogaster* subspecies groups^13,27^. In *D. melanogaster*, pulse songs with conspecific IPI and Kyriacou & Hall (KH) cycles (periodic cycling of mean IPI) increase females’ mating receptivity more than those with heterospecific features, suggesting that IPI is a critical parameter of the courtship song for female receptivity; on the other hand, females have been reported to be unlikely to discriminate the IPF of pulse songs^28–32^ (but see Deutsch et al.^29^). This further suggests that *D. melanogaster* has a neural circuit that discriminates the conspecific IPI of pulse songs.

*D. simulans*, the closest relative of *D. melanogaster*, serves as an exceptional model for evolutionary studies^33–36^. These two species diverged 2.5–3.4 million years ago and are reproductively isolated^37,38^. In each species, whole-genome sequences, as well as transgenics and genome-editing techniques, are available^39,40^. Comparative studies using these two species have revealed responsible genomic regions, relevant genes, and neural functions for phenotypic divergences^35,36^. Recently, the introduction of regulatory sequences that induce gene expressions in specific neurons from *D. melanogaster* into *D. simulans* has allowed reproducible and precise interspecific comparison of homologous interneurons^36^. These features, combined with the expanding knowledge of neuronal circuitry in *D. melanogaster*^41,42^, have advanced *D. simulans* as a compelling model for understanding sensory-processing evolution at the neural circuit level.

Like *D. melanogaster*, males of *D. simulans* court females with a pulse song. However, the song characteristics, namely IPI and IPF, are significantly different between the two species: IPI and IPF are ~ 55 ms and ~ 320 Hz in *D. simulans*, but ~ 35 ms and ~ 170 Hz in *D. melanogaster*, respectively^13,27^. *D. simulans* females also increase their receptivity when exposed to a pulse song with a conspecific IPI comparatively more than those with heterospecific IPIs^6^. Although the song parameters used in previous reports included both species-specific mean IPIs and their KH cycles, IPI preference, a behavioral outcome of auditory sensory processing, is still implied to have diverged between the two closely related species, with *D. simulans* potentially preferring longer IPIs than *D. melanogaster.* This suggests that the auditory neural circuits that specify IPI preference are also diversified between the two species, but no comparative studies on the neural circuits that process song information have been conducted.

In *D. melanogaster*, sound is detected by the Johnston’s organ (JO), the site of auditory mechanotransduction located at the antennal ear^43^. Mechanosensory neurons in the JO, denoted as JO neurons, are the primary neurons of the auditory pathway of flies^20,44,45^. Song information is transmitted mainly from a subset of JO neurons (subgroup-B neurons; JO-B neurons) to the major secondary auditory neurons, AMMC-B1 neurons, which then relay song information to higher-order neurons^46^. Transmissions from JO-B neurons to AMMC-B1 neurons are the first step for IPI information processing in the auditory neural circuit of *D. melanogaster*; while songs with shorter IPIs generate stronger responses in JO-B neurons, the response of AMMC-B1 neurons to the short-IPI song (i.e., 15-ms IPI) is significantly attenuated, thereby shifting its response peak to a longer IPI song (i.e., 25-ms IPI)^31^. However, whether this first step of information processing of *D. melanogaster* is conserved or diversified from its sister species *D. simulans* has not been verified.

In this study, we first systematically compared the behavioral responses to artificial pulse songs with a series of IPIs between *D. melanogaster* and *D. simulans* females*.* Next, to draw a parallel between the early-stage auditory neural circuits, the morphology and neurotransmitter of JO neurons and AMMC-B1 neurons of the two species were characterized. After frequency characteristics of AMMC-B1 were compared, we contrasted neural responses of AMMC-B1 neurons to artificial pulse songs with different IPIs between the species. Our findings indicate an overall conserved, though still slightly divergent, architecture of the early-stage auditory neural circuit between the closely related species.

## Results

### *D. melanogaster* and *D. simulans* exhibit species-specific IPI preferences at a behavioral level

**Figure 1:**
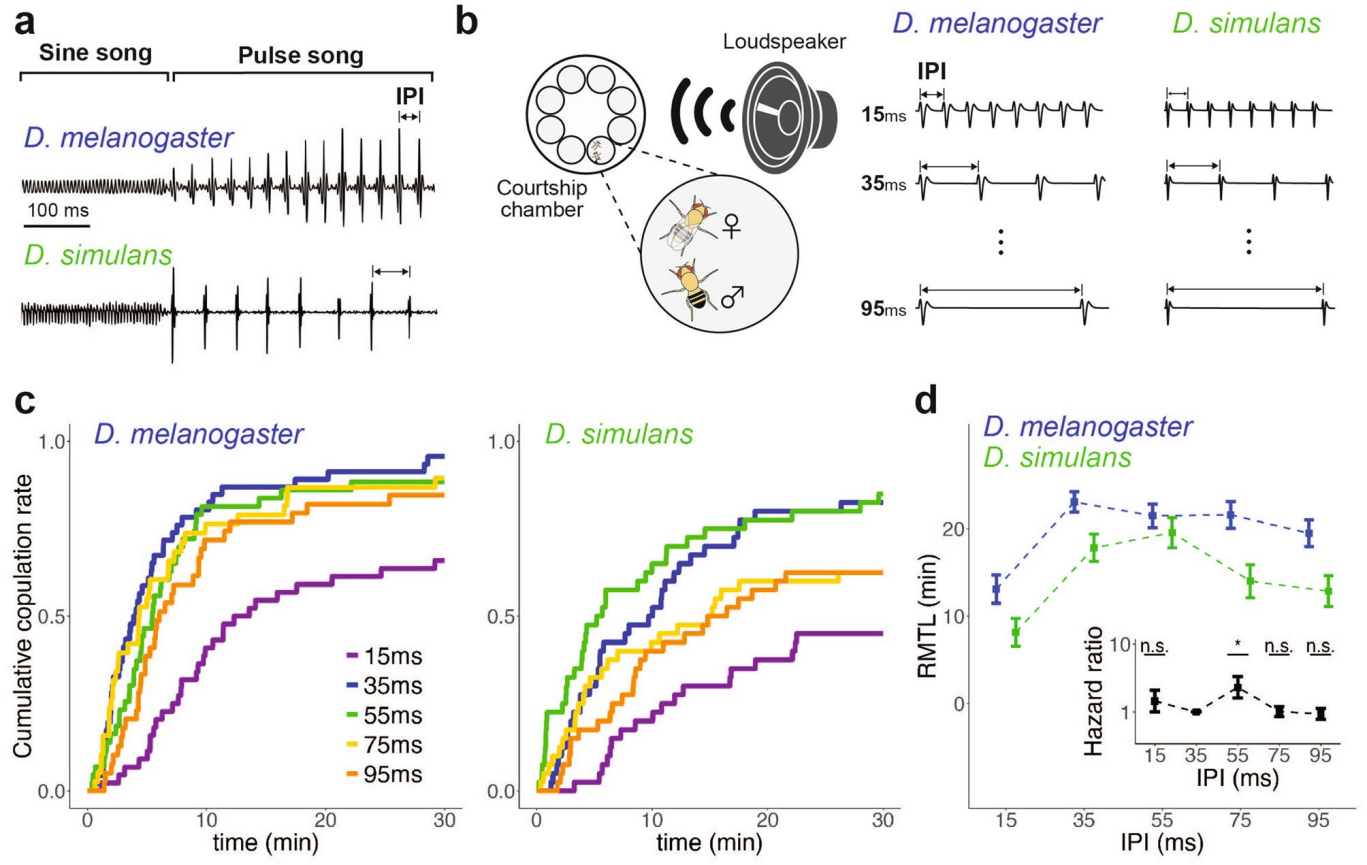
Behavioral responses of two *Drosophila* species to artificial songs with varying IPIs*.* (**a**) Courtship songs of *D. melanogaster* and *D. simulans.* The song is comprised of sine and pulse songs. Inter-pulse intervals (IPIs) of the pulse song are ~ 35 ms in *D. melanogaster* and ~ 55 ms in *D. simulans.* Modified from Kamikouchi and Ishikawa^21^. (**b**) Female copulation assay. An artificial pulse song (Right) was played via a loudspeaker to fly pairs in a courtship chamber (Left). Pulse songs designed with a conspecific intra-pulse frequency (IPF; the principal frequency component of pulses, see Supplementary Fig. S1) were used. Modified from Yamada et al.^31^. (**c**) Cumulative copulation rates during exposure to artificial pulse songs with different IPIs. Left, *D. melanogaster.* Right, *D. simulan.* n = 38–46 pairs per stimulus. (**d**) Restricted mean time lost (RMTL) of cumulative copulation rate for each song. Square dots and error bars represent the average of RMTL and standard errors, respectively. Inset shows hazard ratio of the interaction between IPI (35 ms and X ms) and species (*D. melanogaster* and *D. simulans*) in cumulative copulation rate (See Supplementary methods). The hazard score for the 35-ms IPI song in *D. melanogaster* was used as a reference. When the Hazard ratio (HR) is greater than 1, an increase of copulation rate in *D. simulans* at the song carrying a given IPI (15, 55, 75, or 95-ms) from that at the 35-ms IPI song is higher than that in *D. melanogaster*. HR = 1.44, 2.30, 1.01, and 0.93, and *p* = 0.538, 0.043, 0.979, and 0.890 for 15, 55, 75, and 95-ms IPIs, respectively (Supplementary Table S1). A time window of 0–7 min after the onset of the experiment was used to maintain proportionality (Supplementary Table S6). Square dots and error bars represent the average and standard errors, respectively. n.s.: *p* > 0.05, *: *p* < 0.05; Cox proportional hazard test.

Females of *D. melanogaster* and *D. simulans* increase their copulation receptivity when they are exposed to a courtship song with a conspecific IPI and KH cycles (Fig. 1a)^28,31^. However, KH cycles have recently been suggested to be aliasing artifacts^47,48^. So far, the behavioral responses of females to various IPI songs without KH cycles were not systematically compared between the two species. To investigate the effect of IPIs without KH cycles on these two species, we compared the courtship receptivity of females exposed to artificial songs with varying IPIs between the two species (Fig. 1b).

In general, *D. simulans* showed slower copulation responses than *D. melanogaster*, reminiscent of a previous study that reported slower auditory behavioral responses in *D. simulans* than in *D. melanogaster* (Fig. 1c)^49^. In *D. melanogaster*, pulse songs with IPIs between 35 and 95 ms induced a high copulation rate, while *D. simulans* highly copulated at 35-ms and 55-ms IPI songs (Fig. 1c, Right).

To summarize the response properties to different IPI songs, we used restricted mean time lost (RMTL) as an indicator to represent the area under the curve of the cumulative copulation rate^50^. In this analysis, a larger RMTL reflects that flies are more likely to copulate. Songs with a 15-ms IPI induced the smallest RMTLs in both species, suggesting that this short-IPI song is the least preferred song (Fig. 1d). The largest RMTL, on the other hand, appeared different between these two species. In *D. melanogaster*, the highest RMTL was found for the 35-ms IPI song and gradually decreased for longer IPI songs. In *D. simulans*, the song with a 55-ms IPI resulted in the largest RMTL, while that with a 35-ms IPI resulted in the second largest. These tendencies matched well with their conspecific IPIs (i.e., 35 ms in *D. melanogaster* and 55 ms in *D. simulans*).

Indeed, statistical analysis using the Cox proportional hazard model clearly indicated that the IPI preference of 55-ms over 35-ms was significantly stronger in *D. simulans* than in *D. melanogaster* (*p* = 0.043; See Supplementary methods, Fig. 1d inset and Supplementary Table S1). These findings in females suggest that mating preferences in both species diverged to show preference towards the IPI of conspecific songs.

### Conserved properties of JO neurons between species

JO neurons in the fly ear contain the primary auditory neurons that relay song information to the brain. A previous study has shown that the response of JO neurons to pulse songs is relatively similar in both *D. melanogaster* and *D. simulans*, implying that these sibling species are equally sensitive to pulse songs of both species at the level of the primary auditory neurons^51^. To examine whether other aspects in the early stage of the fly auditory neural circuit are also conserved, we initiated a systematic comparison of song-relay neurons between females of the two species. First, we compared the number, as well as the neurotransmitter and projection patterns, of JO neurons.

**Figure 2:**
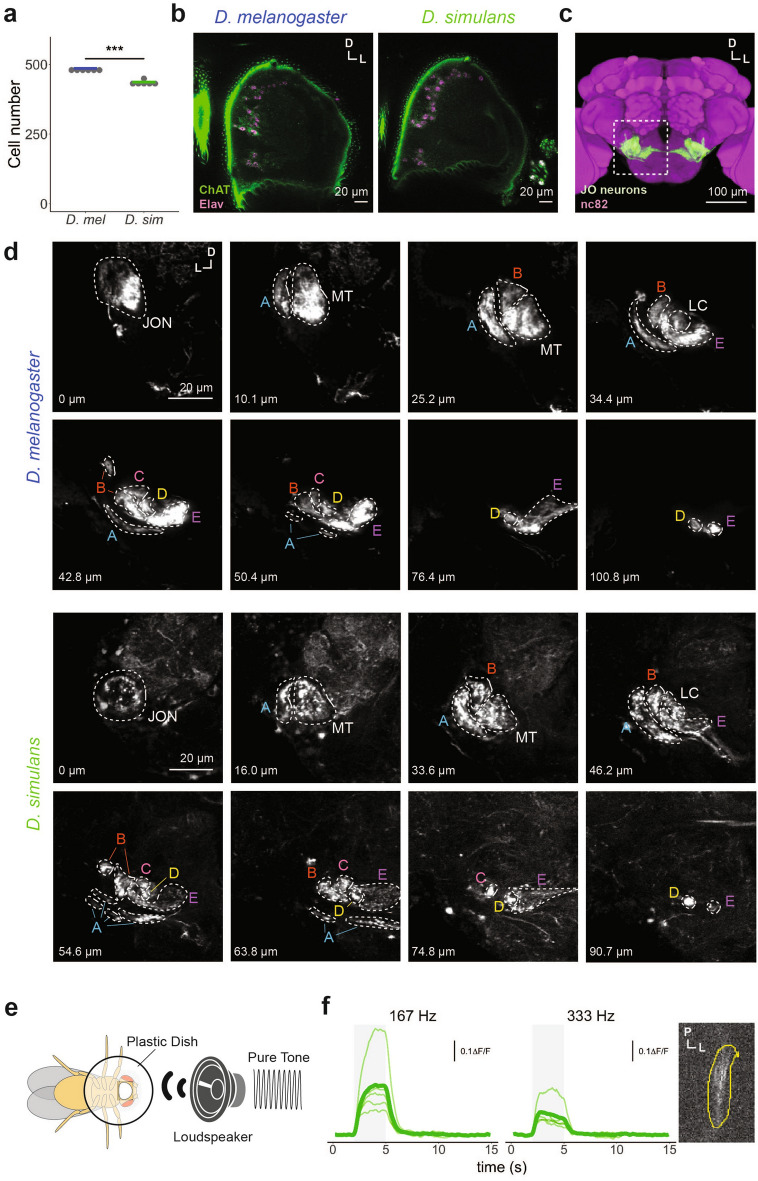
Anatomy, neurotransmitter patterns, and sound responses of JO neurons in *D. melanogaster* and *D. simulans.* (**a**) JO-neuron cell number. Gray dots and colored crossbar indicate the values of each sample and median, respectively. ***p < 0.001; ART ANOVA. (**b**) ChAT signals in JO. Frontal confocal sections of JO within the second antennal segment are shown. Cell bodies of JO neurons were labeled with anti-Elav antibodies (magenta). JO neurons in both species were labeled with anti-ChAT antibodies (green). *D* Dorsal, *L* Lateral. (**c**) JO neuron axons innervating the AMMC region of the brain. JO neuron axons that express GFP by *nanchung-GAL4* driver of *D. melanogaster* are shown in green. Magenta signals show neuropil visualized using the nc82 antibody. A white dashed square area is shown in (**d**). (**d**) Frontal confocal sections of the AMMC along the trajectory of JO neuron axons in *D. melanogaster* (top) and *D. simulans* (bottom) females*.* The fluorescent signals surrounded by dotted lines, which indicate the neurites projecting to the AMMC, are determined to be the axons of JO neurons (see “Methods”). *D* Dorsal, *L* Lateral. *JON* JO neurons, *MT* main trunk, *LC* lateral core region, *A* zone A, *B* zone B, *C* zone C, *D* zone D, *E* zone E. The depth (µm) from the most frontal section was annotated at the bottom left of each panel. (**e**) Experimental setup of calcium imaging. (**f**) Calcium responses of JO neuron axons to pure tones in *D. simulans* (Left, Middle). The sound stimulus, 167-Hz or 333-Hz pure tone, lasted 3 s (shaded gray area). Thin and bold lines show time traces of the response in each individual and the average of all individuals, respectively. The ROI (outlined in yellow) was set at the axon bundle of JO neurons where the fluorescent signals are observable during the sound stimulus (Right). *P* Posterior, *L* Lateral.

To identify cell bodies of JO neurons in both species, we labeled neuronal cell bodies via a rat anti-Elav antibody. We judged labeled cell bodies as forming a cluster in the a2 segment as the cell bodies of JO neurons. We excluded cell bodies located beneath the external sensory bristles on the a2 from cell counting, as these neuronal cells belong to the external sensory organ^52^. In *D. melanogaster* females, the number of JO neurons was found to be 481 ± 6.3 (mean ± SD, n = 6), in accordance with a previous report^52^. *D. simulans* females had 435 ± 9.4 JO neurons (mean ± SD, n = 6), ~ 8% fewer than *D. melanogaster* females (Fig. 2a). We found that most JO neurons of *D. simulans* were positively labeled with anti-Choline-acetyltransferase (ChAT) antibodies (Fig. 2b, Supplementary Fig. S2a) indicating they are presumably cholinergic, as reported in *D. melanogaster*^53–55^.

In *D. melanogaster*, JO neurons are anatomically and functionally divided into five subgroups, subgroups A to E^20,52^ (but see Hampel et al.^56^ for subgroup-F JO neurons). Axons of JO neurons innervate five zones in the antennal mechanosensory and motor center (AMMC) in the brain (Fig. 2c), in which each zone receives input from one of the five subgroups of JO neurons^52^. We compared the zonal organization of JO neuron axons between the species by neural tracing (See “Methods”)^52^. The bifurcation patterns of the labeled projections, which innervate five zones in the AMMC in the *D. melanogaster* brain, were similar to those reported previously for JO neurons^52^, validating our tracing method (Fig. 2d).

Neural tracing in *D. simulans* females revealed that labeled axons also projected to the five zones in the AMMC (Fig. 2d). These axons enter the brain from the antennal nerve (AN) as a single bundle and then bifurcate into zone A and the main trunk (MT). The MT separates off the second lateral bundle to form zone B, and further bifurcates to form zone E and the lateral core region (LC). LC branches off into zones C and D, and finally, zone D reaches the most posterior part of the brain (Fig. 2d). These bifurcation patterns were similar to those observed in *D. melanogaster* (Fig. 2d). Taken together, although the numbers of JO neurons are slightly different*,* the neurotransmitter and axonal projection patterns of JO neurons into five zones are conserved between the species.

Next, we examined the auditory neural response of *D. simulans* JO neurons by utilizing in vivo calcium imaging*.* To label JO neurons genetically, we generated a *D. simulans nanchung-GAL4* strain by introducing a 555 kb upstream sequence of the *nanchung* gene of *D. simulans*, which is homologous to the sequence labeling most JO neurons in *D. melanogaster* (a.k.a. *F-GAL4,* see Kim et al.^57^ and Supplementary methods). To observe the projection patterns of the labeled neurons in the newly generated strain, we performed immunolabeling with an anti-GFP antibody. We found the *D. simulans nanchung-GAL4* labeled only a subset of JO neurons, which innervate all zones in the AMMC except zone D (Supplementary Fig. S2b,c).

To investigate the auditory response of *D. simulans* JO neurons, we conducted calcium imaging using GCaMP6f^58^. In *D. melanogaster*, subgroups A and B of JO neurons (JO-A and JO-B neurons respectively) comprise the major auditory sensory neurons, among which JO-B neurons are a dominant subgroup involved in the song-relay pathway^20,59^. To record the calcium responses of *D. simulans* JO-B neurites, the AMMC zone B was focused on as in a previous report of *D. melanogaster*^31^. However, we found that *D. simulans nanchung-GAL4* showed low-intensity GCaMP signals compared to the *D. melanogaster* counterpart, and the AMMC zone B, as well as other zonal structures comprised of the axons of JO neuron subgroups, were difficult to identify with a GCaMP signal (Supplementary Fig. S2d). This sparse and weak labeling of *D. simulans nanchung-GAL4* precluded an interspecific comparison of response properties specific to JO-B neurons*.*

Despite this limitation, we quantified the auditory responses of a subset of JO neuron axons, in which the GCaMP fluorescence during a sound stimulus was detectable (Fig. 2e,f). The axons of labeled subset showed robust calcium increases in response to pure tones (Fig. 2f). Although we were not able to assign the observed neural responses to specific subgroup(s) of JO neurons, our result indicated that the neurons labeled in the *D. simulans nanchung-GAL4* strain at least responded to acoustic stimuli.

### Conserved properties of AMMC-B1 neurons between species

In *D. melanogaster*, JO-B neurons transmit auditory information to AMMC-B1 neurons, the key secondary auditory neurons in the brain for processing song information^20,31,60^. Silencing of the neural activity of AMMC-B1 neurons in previous studies has revealed their requirements for behavioral responses to courtship songs in females^31,61^. To compare the morphology of AMMC-B1 neurons between the species, we labeled *D. simulans* AMMC-B1 neurons with *R49F09-GAL4*, which was previously used to label AMMC-B1 neurons in *D. melanogaster*^61,62^.

**Figure 3:**
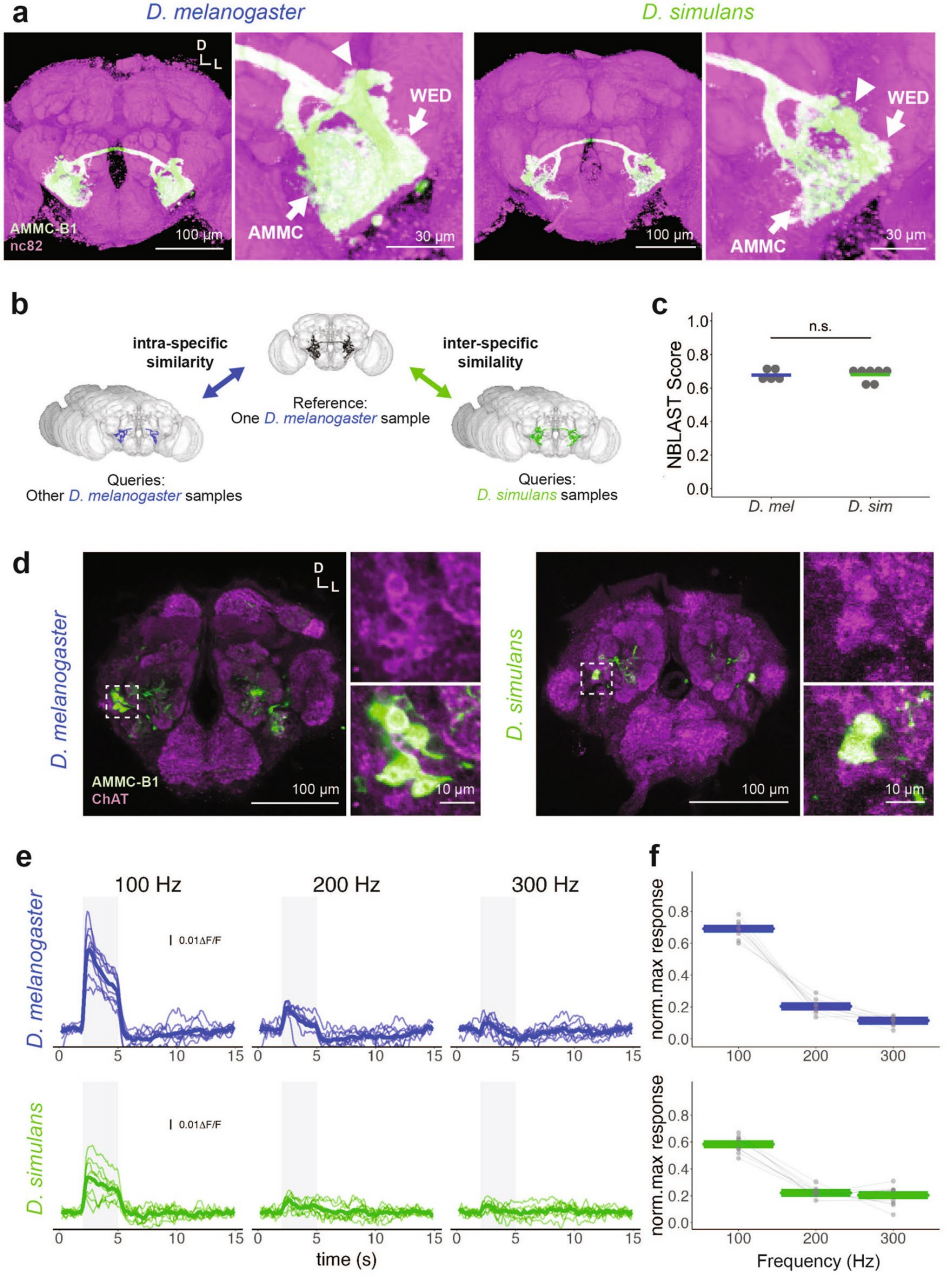
Anatomy, neurotransmitter patterns, and frequency characteristics of AMMC-B1 neurons in *D. melanogaster* and *D. simulans*. (**a**) Morphology of female AMMC-B1 neurons. GFP was expressed in AMMC-B1 neurons by *R49F09-GAL4* driver (green). Magenta signals show nc82 signals. White arrowheads indicate cell bodies of AMMC-B1 neurons. Arrows represent neurites projecting to the AMMC and WED. *D* Dorsal, *L* Lateral. (**b**) Schematic of the comparison of NBLAST scores. One *D. melanogaster* sample was set as the reference (top, dark gray). To quantify the intra-specific similarity of the morphology of AMMC-B1, seven other *D. melanogaster* samples were set as queries (Left, blue). To analyze interspecific similarities, five *D. simulans* samples were set as queries (Right, green). (**c**) NBLAST score of AMMC-B1 neurons of *D. melanogaster* (blue) and *D. simulans* (green). n.s.: *p* > 0.05; ART ANOVA. Gray dots and colored crossbar indicate the values of each sample and median, respectively. (**d**) AMMC-B1 neurons labeled with anti-ChAT antibodies. Green, AMMC-B1 neurons labeled by *R49F09-GAL4* drivers; magenta, ChAT signals. Dashed square areas were magnified in the right panels. *D* Dorsal, *L* Lateral. (**e**) Time traces of AMMC-B1 calcium response to pure tones in *D. melanogaster* (top) and *D. simulans* (bottom)*.* The sound stimulus lasted 3 s (shaded gray area). Thin lines show time traces of the response in each individual. Bold lines represent the average of all individuals. (**f**) Normalized peak responses of AMMC-B1 neurons to pure tones of different frequencies in *D. melanogaster* (top) and *D. simulans* (bottom)*.* Colored crossbars and gray dots represent median and individual data, respectively. Dots of the same individuals are connected with gray lines.

*R49F09-GAL4* is known to label two types of neurons in the *D. melanogaster* brain (Supplementary Fig. S3)^42^. One type is AMMC-B1 neurons, bilateral neurons connecting the AMMC and wedge (WED) of both hemispheres (Fig. 3a, Left; Supplementary Fig. S3)^60^. The other is unilateral neurons (non-AMMC-B1 neurons) that project to the antennal lobe and lateral horn (Supplementary Fig. S3), which resemble olfactory ventral projection neurons^63^. *R49F09-GAL4* in *D. simulans* labeled morphologically similar neurons to both AMMC-B1 and non-AMMC-B1 neurons labeled in *D. melanogaster R49F09-GAL4* (Fig. 3a, Right; Supplementary Fig. S3)*.* We thus concluded that the introduction of *R49F09-GAL4* in *D. simulans* labeled the homologous set of neurons to their *D. melanogaster* counterparts.

The labeled cell bodies of *D. simulans* AMMC-B1 neurons were located dorsolateral to the antennal lobes, as for *D. melanogaster* AMMC-B1 neurons (Fig. 3a). These numbered five to eight in *D. simulans* and nine to twelve in *D. melanogaster* (N = 8 for *D. simulans*, N = 6 for *D. melanogaster*)*.* The neurites of *D. simulans* AMMC-B1 neurons projected to two neuropils, the AMMC and WED, which AMMC-B1 neurons of *D. melanogaster* also project to (Fig. 3a)^60^*.* To quantify the morphological similarity of AMMC-B1 neurons between the species, we compared the NBLAST scores within and between species (see Supplementary methods)^64^. We set the AMMC-B1 neurons of a single *D. melanogaster* individual as a reference (Fig. 3b), and then set the AMMC-B1 of other *D. melanogaster* or *D. simulans* individuals as queries to obtain the NBLAST scores, each of which indicates the intra- and inter-specific similarity, respectively. The NBLAST score in *D. melanogaster* was 0.67 ± 0.015 (N = 7), while the score in *D. simulans* was 0.68 ± 0.017 (N = 5), with no statistical difference being found between the species (p = 0.82, ART ANOVA, Fig. 3c). These results indicate that the morphology of AMMC-B1, at least as labeled in *R49F09-GAL4,* is conserved between the species. A previous study reported that AMMC-B1 neurons in *D. melanogaster* are cholinergic^51^. AMMC-B1 neurons in *D. simulans* were labeled with anti-ChAT antibodies (Fig. 3d), indicating that cholinergic properties are presumably shared between the species.

To examine the auditory responsiveness of AMMC-B1 neurons of *D. simulans* females, we monitored their calcium responses to pure tones ranging from 100 to 300 Hz. Lower-frequency sounds evoked higher responses of AMMC-B1 neurons in both species (Fig. 3e,f; *p* = 0.029, generalized linear model, Supplementary Table S2), with AMMC-B1 neurons barely responding to tones of 300 Hz. The interaction between frequency and species was slightly but significantly different (Species × Frequency; estimate = 3.30E−03, *p* = 5.587E−03), indicating that the preference for the lower frequency sound was significantly stronger in *D. melanogaster* than in *D. simulans.* Taken together, the basic properties of *D. simulans* AMMC-B1 neurons, such as morphology and neurotransmitter distributions, resemble those of *D. melanogaster* AMMC-B1 neurons, though there are some species-specific differences in the frequency characteristics of sound responses.

### Conserved IPI preference of AMMC-B1 neurons with some diversification between species

**Figure 4:**
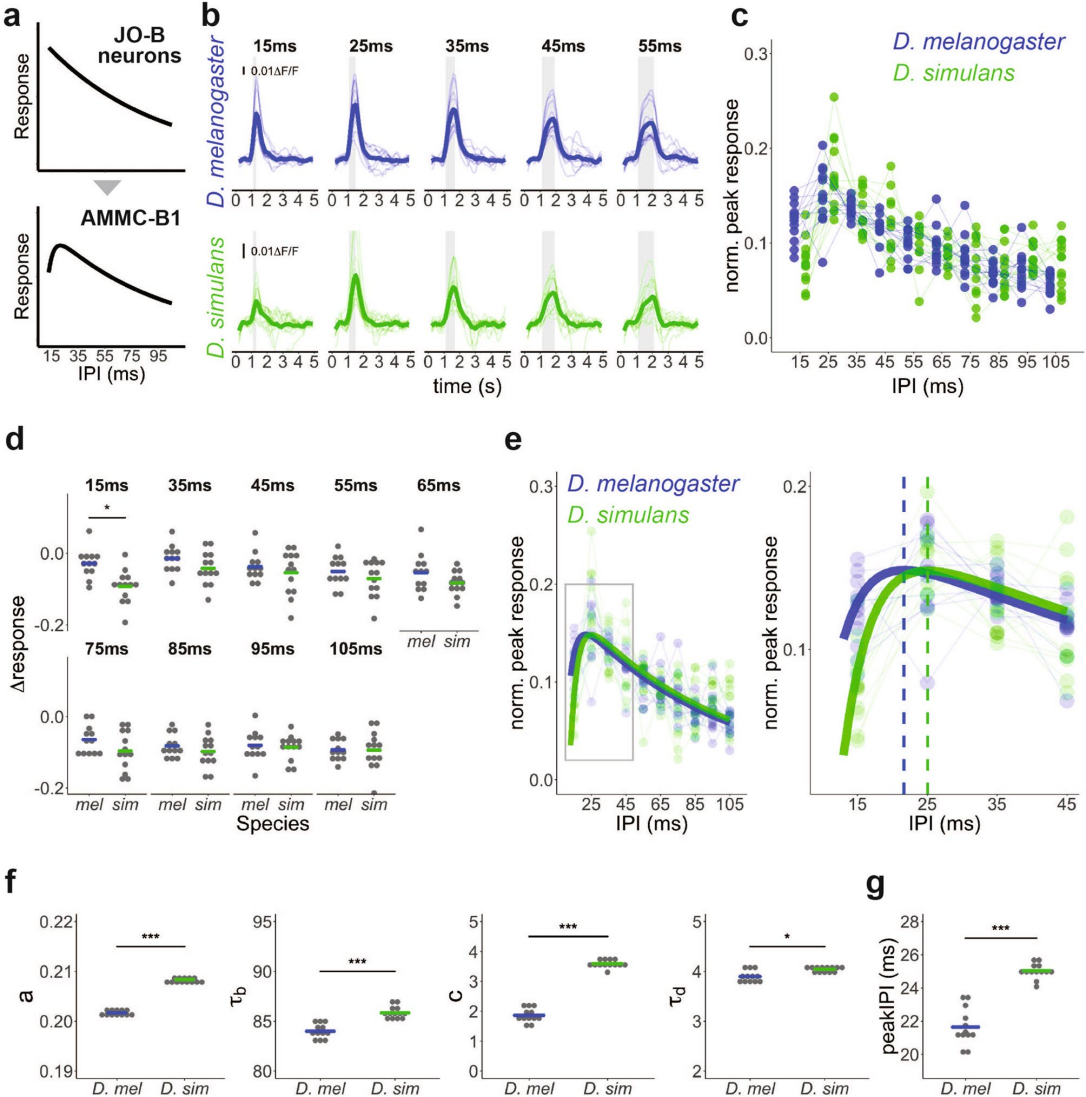
Interspecific comparison of IPI response properties of AMMC-B1 neurons. (**a**) IPI response properties of JO-B and AMMC-B1 neurons in *D. melanogaster*. Modified from Yamada et al.^31^. (**b**) Time traces of AMMC-B1 responses to artificial pulse songs with different IPIs. Responses to songs with 15–55 ms IPIs in *D. melanogaster* (blue) and *D. simulans* (green) are shown (See Supplementary Fig. S4 for all IPI data). Sound stimulus was comprised of 20 pulses for each IPI song (shaded gray area). Pulse songs were designed with a conspecific IPF (see Supplementary Fig. S1). Thin and bold lines show time traces of the response in each individual and the average of all individuals, respectively. (**c**) Normalized peak responses of AMMC-B1 neurons to pulse songs in *D. melanogaster* (blue) and *D. simulans* (green). Dots show normalized peak responses in each individual, where dots of the same individuals are connected with lines. (**d**) Differences from normalized peak responses to pulse song with 25-ms IPI (i.e., ∆responses) in *D. melanogaster* (blue) and *D. simulan*s (green). *p < 0.05; two-tailed *t*-test with Bonferroni correction. Crossbars show median and dots represent individual data. (**e**) Normalized peak responses fitted with Bayesian hierarchical model in *D. melanogaster* (blue) and *D. simulans* (green). Solid smooth lines represent fitted curves. Dots show normalized peak responses in each individual. Dots of the same individuals are connected with thin lines. Left: an overview of the fitting curve. Right: magnified view of short IPI range (left panel grey square). Dashed lines represent IPI where response peaks in *D. melanogaster* (blue) and *D. simulans* models (green), respectively. (**f**) Parameter values obtained from the fitted functions of *D. melanogaster* (blue) and *D. simulans* (green). Dots and crossbars represent the estimated parameters of fitting curves for each individual and all individuals, respectively. *p < 0.05, ***p < 0.001; Exact Wilcoxon rank sum test. (**g**) IPI at peak of individual fitting curve (See Supplementary Fig. S4) in *D. melanogaster* (blue) and *D. simulans* (green). Dots and crossbars indicate the estimated peak IPI of fitting curves for each individual and all individuals, respectively. ***p < 0.001; Exact Wilcoxon rank sum test.

Next, we investigated the responses of AMMC-B1 neurons to pulse songs. In *D. melanogaster*, pulse songs with shorter IPIs elicit stronger responses in JO-B neurons when testing songs with IPIs in the range of 15 to 105 ms (Fig. 4a)^31^. In contrast, AMMC-B1 neurons, which receive auditory inputs mainly from JO-B neurons, exhibit a typical reduction in response at 15-ms IPI (Fig. 4a)^31^. The transformation of IPI selectivity from JO-B neurons to AMMC-B1 neurons is thus regarded as the first step of IPI information processing in *D. melanogaster*. To examine whether the information processing at this level is conserved between the species, we compared the response properties of AMMC-B1 neurons to artificial pulse songs with various IPIs (ranging from 15 to 105 ms). AMMC-B1 neurons of both species showed increased calcium responses to these artificial pulse songs (Fig. 4b, Supplementary Fig. S4).

To investigate the IPI response properties of AMMC-B1 neurons, the normalized peak response, the highest response during the pulse song exposure (i.e., peak response) normalized by the summation of peak responses at all IPIs, was used as an indicator (see Supplementary methods). Our analysis revealed the IPI response properties of AMMC-B1 neurons with asymmetrical band-pass characteristics in both species (Fig. 4c). Among the tested pulse songs with IPIs from 15 to 105 ms in 10 ms increments, the highest responses were observed at 25-ms IPI in both species. The decays of the response from the 25-ms IPI to the shorter IPI were apparently steeper than that to the longer IPIs (Fig. 4c). These overall response patterns were consistent with previous reports in *D. melanogaster*^30,31^ and similar between the species.

Despite such overall similarity, a slight but significant difference was detected in the responses to the 15-ms IPI song. When we compared the response drops from the response at 25-ms IPI to other IPIs (defined as ∆responses hereafter) between the species, the ∆responses only at 15-ms IPI were significantly lower in *D. simulans* than in *D. melanogaster* (*p* = 0.01 at 15-ms, Fig. 4d)*.* This indicates that the decrease in response of AMMC-B1 neurons to short IPI stimuli in *D. simulans* is sharper than that in *D. melanogaster.* Notably, a difference in the IPFs of pulse songs had no significant effect on the IPI response properties of AMMC-B1 neurons (*p* = 0.113 at 15 ms, *p* = 0.990 at 35 ms, Supplementary Fig. S4).

In *D. melanogaster*, AMMC-B1 neurons receive both excitatory and inhibitory inputs (Supplementary Fig. S5)^31^. Inspired by this circuitry information, we fitted the normalized peak calcium responses of AMMC-B1 neurons to a function comprising two types of decay curves by using a hierarchical Bayesian model (Fig. 4e; Supplementary Fig. S4, see Supplementary methods). The fit function is described by

$$y=a{e}^{-x/{\tau }_{b}}-c{e}^{-x/{\tau }_{d}}$$

in which one decay curve, $c{e}^{-x/{\tau }_{d}}$, is deducted from another curve, $a{e}^{-x/{\tau }_{b}}$. The parameters *a* and *c* are the intercept values of each decay curve and *τ*_*b*_ and *τ*_*d*_ are the time constants of each exponential decay. We estimated the fitting function for each individual, and compared the estimated parameters and peaks between the species (Fig. 4f,g; Table 1). The fitting function faithfully recapitulated the actual values (Fig. 4e; Supplementary Fig. S4), and the estimated parameters were robust even when varying initial parameter values for fitting from 0.01 to 1 (data not shown). All the parameters in the fitting function were significantly larger in *D. simulans* than in *D. melanogaster* (*p* = 3.85E−07 at *a*, *p* = 1.54E−06 at *τ*_*b*_*,* *p* = 3.85E−07 at *c*, *p* = 0.011 at *τ*_*d*_*,* Fig. 4f)*.* Of all tested parameters, parameter *c* yielded the largest interspecific differences between the two species (*D. simulans* / *D. melanogaster* =  ~ 1.925; Fig. 4f; Table 1). This result predicts that, at least in the range of parameter values estimated here, *D. simulans* AMMC-B1 receives a more profound reduction of neural responses to the short IPI song than *D. melanogaster* AMMC-B1 (Supplementary Fig. S5).

**Table 1:** Parameters of fit function.

Species	ID	a	tau_b	c	tau_d	peak
*D. melanogaster*	Average	0.2017	83.9797	1.8644	3.8980	21.6406
	1	0.2013	84.0215	1.7965	3.8674	21.3527
	2	0.2009	85.0849	2.1112	4.0494	22.9484
	3	0.2021	83.7082	1.9231	3.8965	21.7417
	4	0.2024	83.0712	1.5545	3.7737	20.2818
	5	0.2014	84.8900	2.2657	4.1005	23.4844
	6	0.2023	83.3108	1.8304	3.8540	21.3208
	7	0.2011	84.3212	1.6926	3.8470	21.0305
	8	0.2013	84.4185	2.0044	3.9433	22.1793
	9	0.2024	82.8497	1.4995	3.7473	20.0106
	10	0.2017	83.7572	1.7758	3.8357	21.1392
	11	0.2013	84.9794	2.1943	4.1031	23.3643
	12	0.2020	83.6037	1.8060	3.8435	21.2328
*D. simulans*	Average	0.2084	85.8339	3.5893	4.0434	25.0429
	1	0.2085	85.0732	3.4764	3.9527	24.3868
	2	0.2088	85.0319	3.3028	3.9362	24.0791
	3	0.2081	86.4459	3.7934	4.1125	25.6852
	4	0.2086	85.8512	3.5614	4.0390	24.9814
	5	0.2083	86.1206	3.6220	4.0441	25.0961
	6	0.2079	85.9810	3.6284	4.0277	25.0185
	7	0.2074	87.1292	3.6964	4.0882	25.4785
	8	0.2083	85.8879	3.5869	4.0156	24.8923
	9	0.2083	85.5537	3.6050	4.0428	25.0496
	10	0.2085	85.3784	3.5757	4.0142	24.8486
	11	0.2088	85.4958	3.6800	4.0551	25.1912
	12	0.2082	85.7921	3.6087	4.0395	25.0463
	13	0.2079	86.7457	3.7893	4.1084	25.6735
*D. simulans/D. melanogaster*		1.0331	1.0221	1.9252	1.0373	1.1572

Parameters of the average and each individual estimated by MCMC are shown. Calcium imaging datasets of *D. melanogaster* (N = 12) and *D. simulans* (N = 13) were used. An ID was assigned to each individual dataset. The ratios of average parameters between species are shown at the bottom (*D. simulans*/*D. melanogaster*).

The estimated peak IPIs (the IPI where the fit function showed peak responses) of these fit functions converged around 25-ms IPI in both species, but were slightly yet significantly larger in *D. simulans* than in *D. melanogaster* (*p* = 3.85E−07, mean: 21.64 ms in *D. melanogaster*, 25.04 ms in *D. simulans*; Fig. 4g). This implied that the IPI selectivity of AMMC-B1 in *D. simulans* shifts toward a longer IPI range than in *D. melanogaster*. In addition, larger *a* and *τ*_*b*_ values in *D. simulans* than in *D. melanogaster* predict that the relative responses of AMMC-B1 to the longer IPI songs are stronger in *D. simulans* than in *D. melanogaster* (Fig. 4f; Supplementary Fig. S5)*.* Although the peak IPI ranges of AMMC-B1 neurons in these species, ~ 25-ms IPI, differed from their behavioral preferences (35-ms IPI for *D. melanogaster* and 55-ms IPI for *D. simulans*), the interspecific differences in the estimated peak IPIs of AMMC-B1 neurons were consistent with the direction of the IPI preference shift at the behavioral level (see Discussion, Fig. 1c,d). Altogether, the calcium imaging and subsequent modeling indicate that the response properties of AMMC-B1 neurons are mostly conserved, although a small diversification between the two species was detected.

## Discussion

Comparisons of acoustic communication in *D. melanogaster* and *D. simulans* serve as an excellent model to understand how sound communication has diversified in short evolutionary time spans^6,13,65–67^. Abundant tools for genetics and connectomics are available in *D. melanogaster*, which enables detailed identification of the neural and molecular bases of sensory processing in this species. The introduction of these tools to *D. simulans*, in turn, allows us to make precise interspecific comparisons of homologous neurons. By combining this with the knowledge of the neural circuit of *D. melanogaster*, we can investigate how sensory processing has evolved at the circuit level. Here we applied this strategy to the auditory neural circuit for the first time, and investigated how the early stages of the circuit in females have been conserved and diversified between the closely related species.

### Divergence of IPI preferences between species

We first demonstrated that the IPI preference of 55-ms over 35-ms was significantly stronger in *D. simulans* than in *D. melanogaster* at the behavioral level*.* Systematic comparison of JO neurons and AMMC-B1 neurons, the primary and a major type of secondary auditory neurons respectively, revealed that morphology and neurotransmitter distributions were conserved between the species. In addition to this overall similarity, we found that AMMC-B1 neurons in *D. simulans* show a small shift in IPI response properties, in which the peak IPI shifted to longer intervals than in *D. melanogaster.* Notably, AMMC-B1 neurons in *D. simulans* have a steeper reduction of neural responses at shorter IPI songs when compared to those in *D. melanogaster*.

Our findings provide strong evidence of conservation with a small diversification in the early stage of the auditory neural circuit, as suggested by a previous study^51^. A small divergence in the early-stage song processing pathway, which we detected in this study, may serve as the first building block of the overall divergence of the entire song-processing pathway. Accordingly, our findings further support the idea of making precise comparisons between *D. melanogaster* and *D. simulans* auditory neural circuits as a powerful system for investigating the neural basis underlying the evolution of acoustic communication.

### Conservation and diversification of JO neurons and AMMC-B1 neurons

The overall characteristics of JO neurons and AMMC-B1 neurons are highly similar between *D. melanogaster* and *D. simulans* despite the difference in the IPI preference at the behavioral level. This suggests that the properties of the early-stage auditory neural circuit are evolutionally conserved in fruit flies. In the zebra finch, neurons that preferentially respond to the conspecific song are located in the latter stage of the auditory neural circuit^68,69^. The combination of these studies and our findings provides some support for the conceptual premise that the properties of peripheral neurons may be evolutionally less flexible than that of higher-order neurons so that species-specificity rarely emerges in the early stage of the neural circuit that processes auditory information.

Despite these conservations, AMMC-B1 still has undeniable diversification in IPI characteristics, especially in the short IPI range. How are the different IPI response properties of AMMC-B1 neurons between the species generated? A hint to answer this question may arise from our knowledge of the auditory neural circuit in *D. melanogaster*, where the IPI response properties of AMMC-B1 neurons are shaped by two GABAergic local interneurons (Supplementary Fig. S5)^31^. These interneurons, AMMC-LN and AMMC-B2, together form a feedforward inhibitory pathway from JO-B neurons to AMMC-B1 neurons to suppress their responses to short (i.e., 15-ms) IPI songs. Considering our prediction that AMMC-B1 neurons receive a more profound reduction of neural responses to short IPI songs in *D. simulans* than in *D. melanogaster*, it is possible that the interspecific difference in IPI selectivity of AMMC-B1 neurons is due to the different properties of, or contribution by, the AMMC-LN and/or AMMC-B2 neurons between the species (Fig. 4; Supplementary Fig. S5).

More specifically, the neural responses or inhibitory outputs of AMMC-LN and/or AMMC-B2 to short IPI songs might be stronger in *D. simulans* than in *D. melanogaster*. Feedforward pathways that generate species-specific interval selectivity have been reported in crickets and fruit flies^31,70^, but no studies have yet demonstrated that they may also contribute to the formation of the interspecific divergence of sound processing. Thus, a comparison of the response properties and neural-circuit architecture of these GABAergic interneurons between the species merits further exploration.

For longer IPIs (55–105 ms IPIs), slight but significant interspecific differences in the parameters were detected in the fitting functions. This finding implies that the relative responses of AMMC-B1 to the longer IPI songs are stronger in *D. simulans* than in *D. melanogaster*. Given our model, the stronger response to the longer IPI songs of AMMC-B1 might be provided by a stronger excitatory input from its upstream JO-B neurons (Supplementary Fig. S5). To evaluate the hypothesis, an interspecific comparison of IPI response properties in JO-B is necessary. Due to the sparse and weak labeling of JO neurons in the *D. simulans nanchung*-*GAL4* strain, however, we were not able to assign the calcium responses to JO-B neurons in this study. To overcome this limitation, a *D. simulans* strain which specifically labels JO-B neurons should be generated.

In the present study, we fit the AMMC-B1 neural responses to a function comprised of two decay curves, in which one decay curve is deducted from another curve. This was inspired by the neural response properties of the excitatory and inhibitory neurons in the feedforward pathway found in *D. melanogaster*^31^, and therefore applying it to *D. simulans* requires an assumption that the basic properties of circuit architecture and neural responses are conserved between the species. The equivalence/similarity of morphology, cell number, and neurotransmitters of JO and AMMC-B1 neurons between the species supports the scenario that basic properties are conserved between species (Fig. 2). To verify these assumptions, however, the neural responses of JO-B neurons, AMMC-B2 neurons, and AMMC-LN of *D. simulans* need to be investigated in the future.

### Frequency tuning of AMMC-B1 neurons

Although frequency characteristics of AMMC-B1 neurons resembled between species, *D. melanogaster* AMMC-B1 neurons had slightly steeper lower-frequency selectivity compared to *D. simulans* (Fig. 3f). This result is consistent with the diversified frequency characteristics (i.e., IPF) of pulse songs; ~ 170 Hz in *D. melanogaster* and ~ 320 Hz in *D. simulans*^13,14,27^. We found however that the differences in IPF, at least for pulses used in this study, had no significant effect on the IPI response properties of *D. melanogaster* AMMC-B1 neurons (Supplementary Fig. S4). This suggests that the IPI preference of AMMC-B1 neurons is possibly independent of the principal frequency component of the individual pulses.

It should be noted, however, that it is not clear if the IPI/IPF interactions of AMMC-B1 neurons are also negligible in *D. simulans*. Further experiments, which systematically compare IPI/IPF interactions in the two species, will shed light on the nature of AMMC-B1 neuronal properties across the entire IPI/IPF landscape.

In *D. melanogaster*, AMMC-B1 neurons are reported to be “pulse preferring neurons” which respond to pulse songs more strongly than pure tones^46^. This current study indicates that AMMC-B1 neurons of *D. simulans* share the pulse preferring characteristics of *D. melanogaster*; *D. simulans* AMMC-B1 neurons showed weak responses to a 300-Hz pure tone (Fig. 3e,f), but clearly responded to pulse songs with a ~ 310-Hz IPF (See “Methods” Fig. 4b; Supplementary Fig. S4). It is not clear whether tuning for pure tone frequencies is reflective of tuning for IPF. The neural mechanisms underlying pulse preferring characteristics, which are shared between species, and IPF characteristics are therefore worthy of further investigation.

Previous studies assigned ~ 60 neurons in each hemisphere as AMMC-B1 neurons, which were further classified into multiple subtypes based on anatomical and functional aspects^46,55,71^. As the number of labeled AMMC-B1 neurons in this study is far less than ~ 60 (nine to twelve in *D. melanogaster* and five to eight in *D. simulans*), the AMMC-B1 neurons analyzed in this study were just a part of the entire AMMC-B1 population. Anatomically, Dorkenwald et al.^71^ classified AMMC-B1 into four subtypes according to their outputs to other cell types in the AMMC. Since this classification relies on information of the synaptic organization of each AMMC-B1 neuron (which requires EM analysis), we were not able to assign AMMC-B1 neurons labeled in our strains to subgroups based on their anatomical classification.

Functionally, AMMC-B1 neurons are classified as low-, middle-, and high-frequency types^55^. The Gal4 driver used in this study (*R49F09-GAL4*) was not subjected to such functional analysis^55^. Our results, however, indicate obvious low-frequency properties and we speculate these neurons are similar to B1 low neurons identified in the aforementioned previous functional study^55^.

### Potential downstream neurons shaping species differences in IPI preference

At the behavioral level, we confirmed a shift of behavioral preference toward longer IPI songs in *D. simulans* as compared to *D. melanogaster* (Fig. 1)*.* Although the shift of neural response of AMMC-B1 observed in *D. simulans* was in the same direction as the shift in their behavioral preferences, it was not large enough to explain the difference observed at the behavioral level. These results suggest that differences in other, possibly downstream, neurons along the auditory neural circuit could further contribute to the interspecific difference in IPI preferences at the behavioral level. Recent studies have reported that vpoEN neurons, which control female virginal plate opening in response to the male courtship song (i.e., female copulatory acceptance) in *D. melanogaster*, preferentially respond to songs with conspecific 35-ms IPI^32,46^. Given that AMMC-B1 is a major type of secondary auditory neuron required for behavioral responses to courtship songs, as shown previously in *D. melanogaster*^31,61^, the neural circuit that receives information from AMMC-B1 and outputs to vpoEN is considered to contribute to further shaping a preference to a conspecific IPI. Indeed, previous studies identified several neurons that connect AMMC-B1 and vpoEN in *D. melanogaster*^32,42,46,61^. If *D. simulans* AMMC-B1 has a homologous downstream neural circuit, vpoEN may show a response preference for a 55-ms IPI in *D. simulans* to shape its behavioral preference for the conspecific IPI. If this is the case, in turn, the interspecific difference of vpoEN may be shaped in the neural circuit between AMMC-B1 and vpoEN. To examine how such a downstream circuit contributes to the interspecific difference in IPI preference at the behavioral level, a comparison of further downstream neurons will be required.

### Future prospects

In this study, we performed precise interspecific comparisons of a homologous auditory neural circuit between closely related species with different sound preferences for the first time. The abundant circuitry information of *D. melanogaster*, together with available genetic tools to label homologous neurons in *D. melanogaster* and *D. simulans*, make this strategy expandable to the entire auditory neural circuit. This study thus lays the groundwork for an unprecedented opportunity to reveal how species-specific auditory information processing has been diversified during the evolutionary process.

## Methods

### *Drosophila* strains

*D. melanogaster* and *D. simulans* were raised on standard yeast-based media at 25°C and 40% to 60% relative humidity under a 12 h light/12 h dark (12 h L/D) cycle. For the female copulation assay shown in Fig. 1, *Canton-S* and *2034 Sim w4 pBac (GCamp6F)5* were used as *D. melanogaster* and *D. simulans* strains, respectively. For counting JO neurons and tracer injection, *Canton-S* and wild type *D. simulans* flies utilised in a previous study^49^ were used for *D. melanogaster* and *D. simulans* strains, respectively. For the copulation assay shown in Supplementary Fig. S1, immunolabeling and calcium imaging, *UAS-GCaMP6f* and *2034 Sim w4 pBac (GCamp6F)5* were used as reporter strains that drive GCaMP6f expression under the UAS promoter, and *D. melanogaster nanchung-GAL4*^57^, *D. simulans nanchung-GAL4*, *D. melanogaster R49F09-GAL4* and *D. simulans R49F09-GAL4* were used as driver lines. Genotypes of flies used for each experiment are listed in Supplementary Table S3. The sample sizes of each experiment were determined according to previous studies^31,72^ and shown in Supplementary Table S4. Detailed information on the *Drosophila* strains is described in Supplementary methods.

### Female copulation assay

The female copulation assay was performed as described previously with minor modifications^31,72^. Both sexes of flies were collected within 8 h after eclosion to ensure their virgin status. Male wings were clipped under ice anesthesia after collection and then kept in individual food vials until experiments were conducted. Females were kept in groups of eight to twelve. Both sexes of flies were maintained at 25°C under a 12 h L/D cycle.

Copulation assays were conducted at 24–26°C and 40–60% relative humidity within 4 h after light onset. Males and females 5 to 7 days after eclosion were used for the assay. A pair of female and male flies were gently aspirated into a chamber (15 mm diameter, 3 mm depth) without anesthesia. The chamber was set above a loudspeaker (Daito Voice AR-10 N, Tokyo Cone Paper MFG. Co. Ltd.) at a distance of 39 mm, and playback of sound stimuli started immediately after aspiration. Fly behaviors were recorded for 35 min at 15 fps with a web camera (Logicool HD Webcam C270). The starting time of copulation was identified manually. Copulation was defined as follows: (1) A male mounts a female for over 5 min, (2) the mounted female reduces her locomotor activity, and (3) opens her wings during the mounting^31,73^.

The sound stimulus and statistical analysis are described in Supplementary methods.

### Tracer injection

Female flies 5 to 10 days after eclosion were fixed ventral side up onto a glass slide. To expose antennal nerves, antennae were removed with sharp forceps. A fluorescent tracer (Dextran, Tetramethylrhodamine, and biotin, 3000 MW, Lysine Fixable; D7162, Invitrogen) dissolved in phosphate-buffered saline (PBS; #T9181, Takara) was placed onto a gap created by removing the antenna. The flies were incubated at 4°C for 2 to 3 h to impregnate the fluorescent tracer into the antennal nerve. The brains were dissected and fixed with 4% paraformaldehyde phosphate buffer solution (#163-201454, FUJIFILM) for 1 h at 4°C. After rinsing with PBS and PBS containing 0.5% Triton X-100 (PBT) (#X100-500ML, Sigma-Aldrich), brains were kept in 50% glycerol (#079-06611, FUJIFILM) in PBS for 30 min and 80% glycerol in deionized water overnight, before being mounted on glass slides to be observed by a confocal microscope. This procedure was replicated for six individual samples, with consistent results identified (data not shown).

### Immunohistochemistry

Immunolabeling of antennae and brains was performed as described previously^60^. Antennae and brains of females 5 to 12 days after eclosion were fixed with 4% paraformaldehyde phosphate buffer solution for 1 h at 4°C, incubated overnight at 4°C in PBT, and incubated with primary and secondary antibodies for 3 days each at 4°C. Samples were rinsed with PBT and PBS, kept in 50% glycerol in PBS for 30 min and 80% glycerol in deionized water overnight, before being mounted on glass slides.

Primary antibodies used in this study were as follows: Rat anti-GFP for enhancing the GCaMP6f signal; Mouse anti-Bruchpilot nc82, to detect synaptic regions in the brain; Rat anti-Elav to label nuclei of JO neurons and AMMC-B1 neurons; Mouse anti-ChAT to visualize ChAT expressed in JO neurons and AMMC-B1 neurons.

The following second antibodies were used: Alexa Fluor 488-conjugated anti-rat IgG, Alexa Fluor 488-conjugated anti-mouse IgG, Alexa Fluor 555-conjugated anti-rat IgG and Alexa Fluor 647-conjugated anti-mouse IgG. This procedure was replicated for at least four individual samples for ChAT staining in JO neurons and AMMC-B1 neurons, with consistent results identified (data not shown). Detailed antibody information is described in Supplementary methods.

### Calcium imaging data acquisition

Calcium imaging was performed as described previously with minor modifications^31^. Six to eight females were collected in each food vial within 8 h after eclosion. They were maintained at 29°C under a 12 h L/D cycle for nine to twelve days after eclosion until the experiments to enhance GCaMP6f expression. Flies were anesthetized on ice and stabilized ventral side up onto an imaging plate using silicon grease (#SH 44 M, Toray). The mouthparts of the fly were removed using fine forceps to open a window to monitor GCaMP fluorescence from the brain. A drop of an adult saline solution with the following components was added to prevent dehydration: 108 mM NaCl, 5 mM KCl, 2 mM CaCl_2_, 8.2 mM MgCl_2_, 4 mM NaHCO_3_, 1 mM NaH_2_PO_4_, 5 mM trehalose, 10 mM sucrose, and 5 mM HEPES, pH 7.5, 265 mOsm^74^. A fluorescent microscope (Axio Imager.A2, Carl Zeiss) equipped with a water-immersion 20 × objective lens [W Achroplan/W N-Achroplan, numerical aperture (NA) = 0.5; Carl Zeiss], a spinning disk confocal head CSU-W1 (Yokogawa), and an OBIS 488 LS laser (Coherent) for excitation at 488 nm was utilized.

To provide sound stimuli, a loudspeaker (Daito Voice AR-10 N, Tokyo Cone Paper) was positioned ∼11 cm from the antenna of the fly. For monitoring calcium responses to pure tones (Figs. 2e,f, 3e,f), 3-s pure tones of the following frequency combinations were randomly applied: 167 and 333 Hz for JO neurons; 100, 200, and 300 Hz for AMMC-B1 neurons.

For measuring the IPI property of AMMC-B1 neurons (Fig. 4, Supplementary Fig. S4), artificial pulse stimuli comprised of 20 pulse trains, which were generated as the pulses used in the female copulation assay, were delivered from the loudspeaker. The IPFs of actual playback sounds were ~ 170 Hz for *D. melanogaster* and ~ 310 Hz for *D. simulans.* These IPFs are within the range of the natural songs of the two species^13^. For each trial, pulse trains with ten kinds of IPI (15, 25, 35, 45, 55, 65, 75, 85, 95, and 105 ms) were randomly applied. For evaluating the effect of IPF on the responses of AMMC-B1 neurons (Supplementary Fig. S4), six kinds of artificial pulse songs comprised of the combinations of two IPFs (~ 170 and ~ 310 Hz, which are in the ranges of *D. melanogaster* and *D. simulans* songs, respectively) and three IPIs (15, 25, and 35 ms) were delivered in a randomized order, in which each of the six pulse sounds carried 20 pulses.

The mean peak-to-peak amplitude of the particle velocity was 47.2 mm/s for both pure tones and pulse songs. This velocity was set to enable monitoring of subtle responses to higher frequency tones (200 Hz and 300 Hz). As our labeled AMMC-B1 neurons showed stronger responses to the lower frequency tone, 40 Hz tone was used as a positive control stimulus at the end of each trial. We also confirmed that the responses to all the other tones (100, 200, 300 Hz, and pulse songs) were not saturated, as all of these stimuli evoked smaller responses than the 40 Hz tone.

Calcium imaging was conducted at 24–26°C and 40–60% relative humidity. For each individual, we measured the calcium response to a series of pulse sounds for 3 trials and pure tones for 4 trials with a > 5 s inter-trial interval between each acoustic stimulus. The fluorescent images were captured at 10 frames/s with an exposure time of 100 ms using an EM-CCD camera (ImagEM, C9100-13; Hamamatsu Photonics) at a spatial resolution of 512 × 512 pixels in water-cooled mode.

### Analysis of calcium imaging data

Calcium-imaging data was analyzed using Python, Fiji^75^, Excel (Microsoft), and R software. Aberrations of serial images due to the animal movement were corrected using a scikit-image library in Python^76,77^.

For the analysis of JO neurons, the region of interest (ROI) was set at the region of the axon bundle of JO neurons where the fluorescent signals are observable during sound stimulus (Fig. 2f, Supplementary Fig. S2). For the analysis of AMMC-B1 neurons, the ROI was set in the dendric region within the AMMC as described in a previous study (Supplementary Fig. S3)^31^. The ROI did not include the projections of the non-AMMC neurons. The mean fluorescence intensity in the ROI was measured for each frame using Fiji. To obtain smooth time traces of calcium fluorescence, moving averages of fluorescence intensity for three frames were used for data analysis.

The decay of fluorescence intensity due to bleaching out was corrected as follows: (1) A linear fit function was estimated by the fluorescence at 0–1 and 4–5 s (for pulse-song stimuli) or 0–2 and 13–15 s (for pure-tone stimuli). (2) The raw fluorescence was corrected by deducting the linear fit function. The decay corrections were performed using the stats package in R (https://stat.ethz.ch/R-manual/R-devel/library/stats/html/00Index.html). The relative fluorescence change (∆*F/F*) of GCaMP6f was used to indicate the calcium response, which was given by

$$\Delta F/F \, = \, \left( {F_{n} {-} \, F_{Base} } \right)/F_{Base}$$

where *F*_*n*_ is the corrected fluorescence intensity at *n* seconds from the sound onset. *F*_*Base*_ is the average of the corrected fluorescence intensity during the 1 s before the stimulus onset. We defined the peak response in each stimulus as the highest ∆*F/F* value obtained during each stimulus period. Detailed methods are described in Supplementary methods.

## Supplementary Information

Supplementary Information.

## Data Availability

All data and R scripts used in the analyses are available on Dryad (http://www.datadryad.org) via (https://doi.org/10.5061/dryad.tdz08kq3f)^78^.
