# Supplementary material for: Evolutionary conservation and diversification of auditory neural circuits that process courtship songs in Drosophila
Source: Sci Rep. 2023 Jan 7;13:383. doi: 10.1038/s41598-022-27349-7 (PMC9825394; doi:10.1038/s41598-022-27349-7)
Supplement: Supplementary file 1 — Supplementary Information. [file 41598_2022_27349_MOESM1_ESM.pdf]

Supplementary Material

Title

Evolutionary conservation and diversification of auditory neural circuits that process courtship songs in *Drosophila*

Authors

Takuro S. Ohashi[1], Yuki Ishikawa[1]*, Takeshi Awasaki[2], Matthew P. Su[1, 3], Yusuke Yoneyama[1], Nao Morimoto[4] and Azusa Kamikouchi[1, 5]*

Author Affiliation

[1] Graduate School of Science, Nagoya University, Nagoya, Aichi, 464-8602, Japan

[2] School of Medicine, Kyorin University, Tokyo, 181-8611, Japan

[3] Institute for Advanced Research, Nagoya University, Nagoya, Aichi, 464-8601, Japan

[4] Institute for Genetic Medicine, Hokkaido University, Sapporo, Hokkaido, 060-0815, Japan

[5] Graduate School of Life Sciences, Tohoku University, Sendai, Miyagi, 980-8577, Japan

Footnotes

* These authors are joint corresponding authors.

Supplementary methods

Resource table

REAGENT or RESOURCE	SOURCE	IDENTIFIER	Additional information
**Plasmid**			
R49F09-GAL4	PMID: 23063364	NA	Gift from Dr. Rubin
D. simulans nanchung-GAL4	This study	NA	Laboratory of Dr. Awasaki
**Genetic reagent (*D. melanogaster*)**			
Canton-S	PMID: 24086330	NA	Gift from Dr. K. Ito
D. melanogaster F-GAL4	PMID: 29691331	NA	Gift from Dr. Changsoo Kim
D. melanogaster R49F09-GAL4	PMID: 23063364	RRID:BDSC_38699	
D. melanogaster UAS-GCaMP6f	Bloomington Drosophila Stock Center	RRID:BDSC_42747	
**Genetic reagent (*D. simulans*)**			
D. simulans wild type	Yoon et al. 2013	N/A	Originally from San Diego Drosophila Species Stock Center
D. simulans nanchung-GAL4	This study	NA	Laboratory of Dr. Kamikouchi
D. simulans R49F09-GAL4	This study	NA	Laboratory of Dr. Kamikouchi
D. simulans UAS-GCaMP6f (2034 Sim w4 pBac (GCamp6F)5)	This study	NA	Gift from Dr. Stern
attP2176 strain	PMID: 28280212	NA	Gift from Dr. Stern
**Software**			

Fiji (version: 2.0.0-rc-69/1.52p)	PMID: 22743772	RRID: SCR_002285	
Audacity (version 2.0)	https://www.audacityteam.org/	RRID: SCR_007198	
VVD Viewer	https://github.com/takashi310/VVD_Viewer/releases	RRID:SCR_021708	
**Tool**			
R (version 4.0.3)	https://www.r-project.org/	RRID: SCR_001905	
Python	https://www.python.org/	RRID:SCR_008394	
Stan	https://mc-stan.org	RRID:SCR_018459	
NBLAST on the fly	PMID: 27373836	RRID:SCR_015884	
**Antibody**			
Rat anti-elav (rat monoclonal; supernatant)	Developmental Studies Hybridoma Bank (DSHB)	RRID: AB_528218	(1:250)
Rat anti-GFP (rat monoclonal)	NACARAI TESQUE, INC	RRID: AB_221569	(1:1000)
Rabbit anti-GFP (rabbit polyclonal)	Invitrogen	#A-11122	(1:1000)
Mouse nc82 (mouse monoclonal; supernatant)	DSHB	RRID: AB_2314866	(1:20)
Mouse anti-ChAT (mouse monoclonal; supernatant)	DSHB	RRID: AB_528122	(1:50)
Goat anti-mouse-Alexa 488 (goat polyclonal)	Thermo Fisher	RRID: AB_2534088	(1:300)
Goat anti-rat-Alexa 488 (goat polyclonal)	Jackson	RRID: AB_2338362	(1:300)
Goat anti-rat-Alexa 555 (goat polyclonal)	Thermo Fisher	RRID: AB_2535855	(1:300)
Goat anti-mouse-Alexa 647 (goat polyclonal)	Thermo Fisher	RRID: AB_2535805	(1:300)

Sound stimulus for copulation assay

For the sound stimulus, we generated artificial pulse songs with five different IPIs (15, 35, 55, 75, and 95 ms), with the duration of each being 3 s, using the Audacity software (Fig. 1b and Supplementary Fig. S1). This 3-s sound file is comprised of a repetition of pulses (1-s of pulse burst) and a subsequent 2-s pause as described previously[1]. To generate the pulse bursts with different IPIs, we interspersed one cycle of sine waves with a variable duration of silence. Since a previous study reported no significant effect of IPF, the frequencies of one cycle of the sine wave, on behavioral responses[2], IPF used to design the artificial pulse songs were 167 Hz for *D. melanogaster* and 333 Hz for *D. simulans* to be consistent with the conspecific range of each species (Supplementary Fig. S1)[3].

The IPFs of the actual playback sounds were ~170 Hz for *D. melanogaster* and ~310 Hz for *D. simulans*, both of which were still in the conspecific range of each species[3]. These IPFs of the playback sounds were estimated from the average time between peak and bottom of the waveform of three recorded pulses. The actual pulses generated by a speaker playback are shown in Supplementary Fig. S1. We played each 3-s sound file repeatedly during the 30-min observation period of the female copulation assay. The mean peak-to-peak amplitude of the resulting particle velocity was 24.8 mm/s.

Statistical analysis of copulation assay

Restricted mean time lost (RMTL)[4] values were calculated using the R 'survRM2' package (https://cran.r-project.org/web/packages/survRM2/index.html). Larger RMTL values indicate that flies were more likely to copulate.

To compare IPI preferences at the behavioral level between the species, we evaluated the interaction of two covariates, IPI and species, on female receptivity (Supplementary Fig. S1). Since RMTL analyses are not applicable for two variables, we utilized the Cox proportional hazard model generated in the R 'survival' package (https://cran.r-project.org/web/packages/survival/index.html). In order to maintain the assumption of time-invariant proportional hazards in two assays shown in Fig. 1 and Supplementary Fig. S1, we divided the observation time into two phases, 0-7 min and 7-30 min, after the onset of the assay (Supplementary Table S1 and S6). Hazard ratios (HRs)

of the interaction between IPI and species represent a species difference in the effect of IPI on the possibility of copulation events. The hazard value for 35 ms IPI in *D. melanogaster* was used as the reference to obtain the HR. HR was calculated for both phases (Supplementary Table S1), with data from only 0-7 min for wild type (Fig. 1d inset) and 7-30 min for calcium imaging strains (Supplementary Fig. S1b inset) included as graphs. When the HR is greater than 1, an increase of copulation rate in *D. simulans* at the song carrying a given IPI (15, 55, 75, or 95 -ms) from that at the 35-ms IPI song is higher than that in *D. melanogaster*.

Confocal microscopy and image processing

Serial optical sections of the antennae and brains were obtained at 0.84-μm (brains) or 0.57-μm (brains and antennae) intervals with an FV1000-D (Fig 2d) or FV1200 (other figures) laser-scanning confocal microscope (Olympus, Tokyo, Japan) equipped with a silicone-oil immersion 30x (brains) or 60x (brains and antennae) Plan-Apochromat objective lens (NA = 1.05 and 1.3, respectively). For the tracer injection experiment, JO neurons were discriminated as neurons that projected to the AMMC in the brain[5]. The zones in the AMMC were identified based on the nc82 labeling pattern. For three-dimensional (3D) image reconstruction, confocal image datasets were processed with the 3D-reconstruction software VVD Viewer. To extract the projection pattern of the AMMC-B1 neurons (Fig 3A), the signals not derived from AMMC-B1 neurons were manually eliminated from the *R49F09-GAL4* images (Supplementary Fig. S3) using VVD Viewer.

The number of JO neurons was counted using EVEA (Excluded-Volume-Embedding Algorithm)[5] with a subsequent manual correction. The number of AMMC-B1 neurons was counted manually. For statistical comparison of cell numbers, we used the ARTool package in R (https://cran.r-project.org/web/packages/ARTool/index.html) [6] to perform aligned rank transform analysis of variance (ART ANOVA) tests.

NBLAST

For comparison of the anatomical characteristics of AMMC-B1 (Fig. 3b), we utilized NBLAST similarity scores[7] following the methodology described on the

following website（http://flybrain.mrc-lmb.cam.ac.uk/si/nblast/www/nblast_desktop/）.

Briefly, image stacks of brains were aligned to a template brain with non-rigid registration using the Computational Morphometry Toolkit[8] (RRID:SCR_002234). The aligned images were reconstructed to 3D images and all neurons except AMMC-B1 were eliminated using VVD Viewer. The extracted neurons were skeletonized with an image processing package Fiji. Subsequently, the skeletonized neurons were vectorized and compared using the nat.nblast R package. One *D. melanogaster* brain was used as a reference and other *D. melanogaster* brains and *D. simulans* brains were analyzed as queries. Scores were statistically evaluated using ART ANOVA.

Generation of *D. simulans* transgenic strains

To generate a *D. simulans nanchung-GAL4* strain, we introduced a plasmid including *nanchung* regulatory sequences followed by a *GAL4* coding sequence.

A 555 kb upstream sequence of the *D. simulans nanchung* gene was cloned and integrated into linearized pBPGUw (Catalog#17575, RRID: Addgene_17575, Addgene) with EcoRI and NaeI using an In-Fusion HD Cloning Kit (Takara)[57].

The primer sequences used to clone the *nanchung* regulatory sequence were as follows:

Forward: TCGTCTTCAAGAATTGAATTCAAAATTCATTTGTAAAAG

Reverse: CGGGCGAGCTCGGCCCCGATCCCGAATTCACTTT

Note: Underlined characters indicate 15-bp extensions that are complementary to the ends of the linearized vector.

The plasmid was introduced by φC31-mediated recombination into the *D. simulans TG-S15 attP* strain (Awasaki et al., in preparation).

To generate a *D. simulans R49F09-GAL4* strain, we transformed the *R49F09-GAL4* plasmid into the *attP2176* strain[9] using φC31-mediated recombination. Injections for generating *D. simulans R49F09-GAL4* were performed by Rainbow Transgenic Flies, Inc.

To label JO neurons in *D. simulans*, we introduced a *nanchung-GAL4* sequence to the *TG-S15 attP* strain, resulting in only a subsection of JO neurons being labeled (Supplementary Fig. S2a). Given that different *attP* landing sites generally drive different

levels and patterns of transgene expression[9,10], the low expression in JO neurons may be due to the genetic environment surrounding the *attP* landing site. As the nucleotide sequence *nanchung* promoter region (555kb upstream sequence of *nanchung* gene) is slightly diversified between *D. melanogaster* and *D. simulans*, it is also possible that species differences in the promoter sequence result in differences in driving *GAL4*-expression level.

<u>Calculation of calcium imaging data</u>

The frequency response properties of AMMC-B1 neurons were assessed using a generalized linear model (GLM) (Fig. 3f). Frequency and species were used for explanatory variables, and normalized peak response was set as a response variable. A gamma distribution was used for error structure, and a logarithmic function was set as the link function.

To evaluate the IPI response properties and IPF effects of AMMC-B1 neurons (Fig. 4d, Supplementary Fig. S4), delta (Δ) response was calculated as the differences between normalized peak responses at 25 ms IPI and other IPIs. Since all datasets were not rejected by Shapiro-Wilk and Bartlett tests, we used a two-tailed t-test with Bonferroni correction for statistical analysis of Δresponses for the IPI response properties, and a pairwise t-test with Bonferroni correction for the IPF effects, respectively. To analyze the response properties for different frequencies (Fig. 3e and 3f), we calculated the normalized peak response as

$$(peak\Delta F/F_{FrequencyX})/\ (peak\Delta F/F_{Total})$$

where peak response when the fly was exposed to a sound of *X*-Hz frequency ($peak\Delta F/F_{FrequencyX}$) was normalized by the summation of peak responses at all frequencies, excluding 40 Hz of pure tones used as positive controls ($peak\Delta F/F_{Total}$). If the fluorescent data were normalized by a single value, the normalized data would fluctuate according to variation of the value used for normalization. Summation levels reduce individual datasets' uneven nature and represent the magnitude of overall responses. Therefore, we utilized this summation rather than a maximum response across stimuli.

To analyze the response properties for different IPIs (Fig. 4), we calculated the

normalized peak response as

$$(peak\Delta F/F_{IPI=X})/\ (peak\Delta F/F_{Total})$$

where peak response with *X*-ms IPI ($peak\Delta F/F_{IPI=X}$) was divided by the summation of peak responses at all IPIs ($peak\Delta F/F_{Total}$). For evaluating the IPF effect of AMMC-B1 neurons (Supplementary Fig. S4), $peak\Delta F/F_{Total}$ of each IPF was calculated separately.

Bayesian hierarchical modeling for fitting

Normalized peak responses were fitted with the function

$$y = ae^{-x/\tau_b} - ce^{-x/\tau_d}$$

using Bayesian hierarchical modeling. We designed a Bayesian network to estimate parameters for individual flies (see Supplementary Fig. S4). Each parameter was estimated using Markov chain Monte Carlo (MCMC) methods in the stan package (Stan Development Team. Stan Modeling Language Users Guide and Reference Manual 2.27. https://mc-stan.org/docs/2_27/stan-users-guide/index.html) via the R interface, Rstan. The initial values of MCMC were set as a = 0.1, b = 0.01, c = 0.1, d = 0.1 (See Supplementary Table S5). R-hat values (diagnostic values to check whether the MCMC has converged or not) of all convergence values were confirmed to be lower than 1.1. We also tested other initial parametric values in a range from 0.1 to 1.0; convergence values were almost unchanged throughout this range, though occasional R-hat values were over 1.1. To compare parameters and peaks of fitted functions (Fig. 4f and 4g), we utilized the Exact Wilcoxon rank sum test with the exactRankTests package in R (https://cran.r-project.org/web/packages/exactRankTests/index.html).

References

1. Yoon, J. *et al.* Selectivity and plasticity in a sound-evoked male-male interaction in *Drosophila*. *PLoS One* 8, (2013).
2. Bennet-Clark, H. C. & Ewing, A. W. Pulse interval as a critical parameter in the courtship song of *Drosophila melanogaster*. *Anim Behav* 17, 755–759 (1969).
3. Riabinina, O., Dai, M., Duke, T. & Albert, J. T. Active process mediates species-specific tuning of *Drosophila* ears. *Curr Biol* 21, 658–664 (2011).
4. Uno, H. *et al.* Moving beyond the hazard ratio in quantifying the between-group difference in survival analysis. *J Clin Oncol* 32, 2380–2385 (2014).
5. Kamikouchi, A., Shimada, T. & Ito, K. Comprehensive classification of the auditory

sensory projections in the brain of the fruit fly *Drosophila melanogaster*. *J Comp Neurol.* **499**, 317–356 (2006).

6. Wobbrock, J. O., Findlater, L., Gergle, D. & Higgins, J. J. The Aligned Rank Transform for nonparametric factorial analyses using only ANOVA procedures. *Proceedings of the ACM Conference on Human Factors in Computing Systems* 143–146 (2011).
7. Costa, M., Manton, J. D., Ostrovsky, A. D., Prohaska, S. & Jefferis, G. S. X. E. NBLAST: Rapid, sensitive comparison of neuronal structure and construction of neuron family databases. *Neuron* **91**, 293–311 (2016).
8. Jefferis, G. S. X. E. *et al.* Comprehensive maps of *Drosophila* higher olfactory centers: Spatially segregated fruit and pheromone representation. *Cell* **128**, 1187–1203 (2007).
9. Stern, D. L. *et al.* Genetic and transgenic reagents for *Drosophila simulans, D. mauritiana, D. yakuba, D. santomea,* and *D. virilis*. *G3-GENES GENOM GENET* **7**, 1339–1347 (2017).
10. Pfeiffer, B. D. *et al.* Refinement of tools for targeted gene expression in Drosophila. *Genetics* **186**, 735–755 (2010).
11. Yamada, D. *et al.* GABAergic local interneurons shape female fruit fly response to mating songs. *J Neurosci* **38**, 4329–4347 (2018).

Supplementary Figures

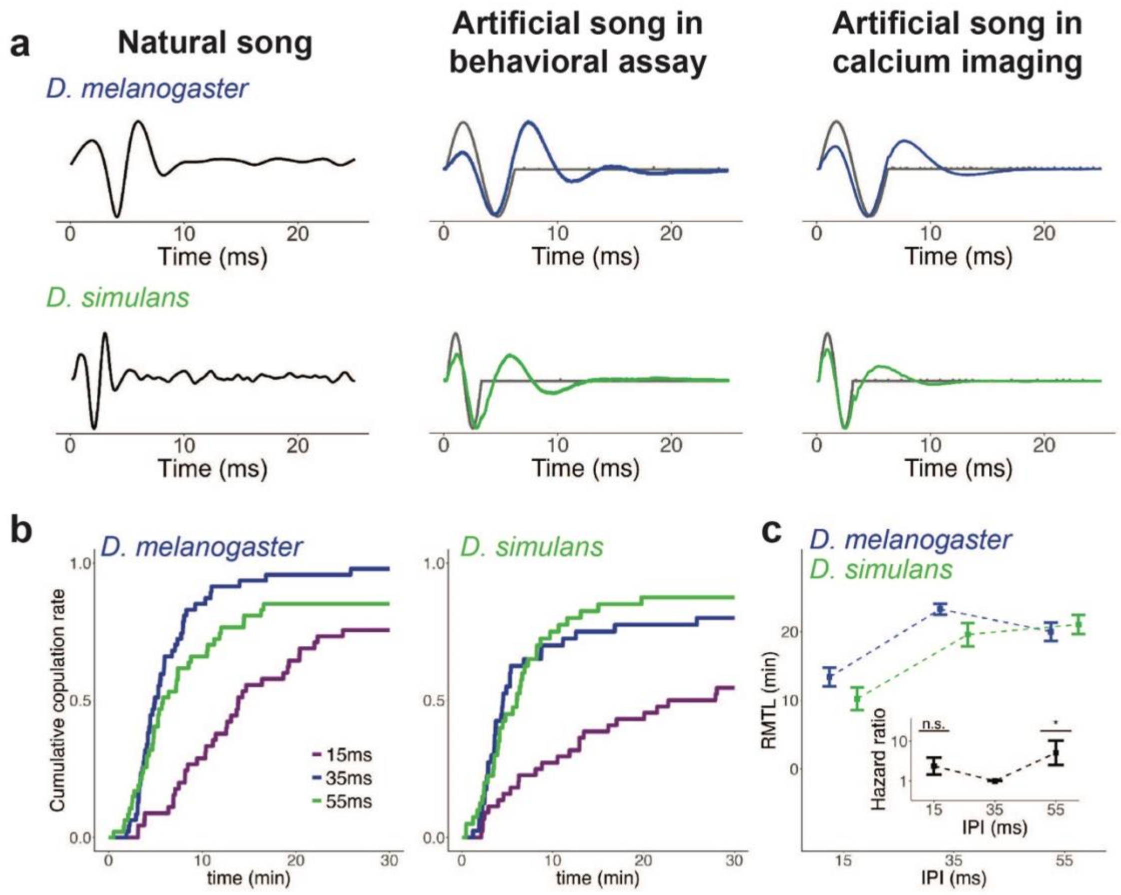

<u>Supplementary Figure S1.</u> Behavioral responses of two *Drosophila* species to artificial songs and their waveforms. (**a**) Sound waveforms of pulse songs. Natural and artificial pulse songs. *D. melanogaster* (top) and *D. simulans* (bottom) are shown. Left: A pulse that comprises natural pulse songs in each species. Middle and Right: An input pulse (one cycle of sine waves and a subsequent silent period are shown) to generate the artificial pulse song used in this study (grey) and its playback sound (colored) recorded with a pressure-gradient microphone (An Emkay NR3158, Knowles Electronics Inc., Itasca, IL) in the behavioral assay (Middle) and calcium imaging (Right). The IPFs for *D. melanogaster* (blue) and *D. simulans* (green) playback songs, emitted by the speaker, were ~170 Hz and ~320 Hz, respectively. Differences between the artificial stimuli generated vs. that recorded were due to speaker response properties. (**b**) Cumulative copulation rates during exposure to artificial pulse songs with different IPIs in strains used for calcium imaging of AMMC-B1 neurons. Left, *D. melanogaster*. Right, *D. simulans.* n = 40 – 47 pairs per stimulus. (**c**) RMTL of cumulative copulation rate for each song. Square dots and error bars represent the average of RMTL and standard errors,

respectively. Inset shows hazard ratio of the interaction between IPI (35 ms and X ms) and species (*D. melanogaster* and *D. simulans*) in cumulative copulation rate. HR = 2.37 and 5.09, and p = 0.144 and 0.011 for 15 and 55 -ms IPIs, respectively (Supplementary Table S1). A time window of 7-30 min after the onset of the experiment was used to maintain proportionality (Supplementary Table S6). Square dots and error bars represent the average and standard errors, respectively. n.s.: $P > 0.05$, *: $P < 0.05$; cox proportional hazard test.

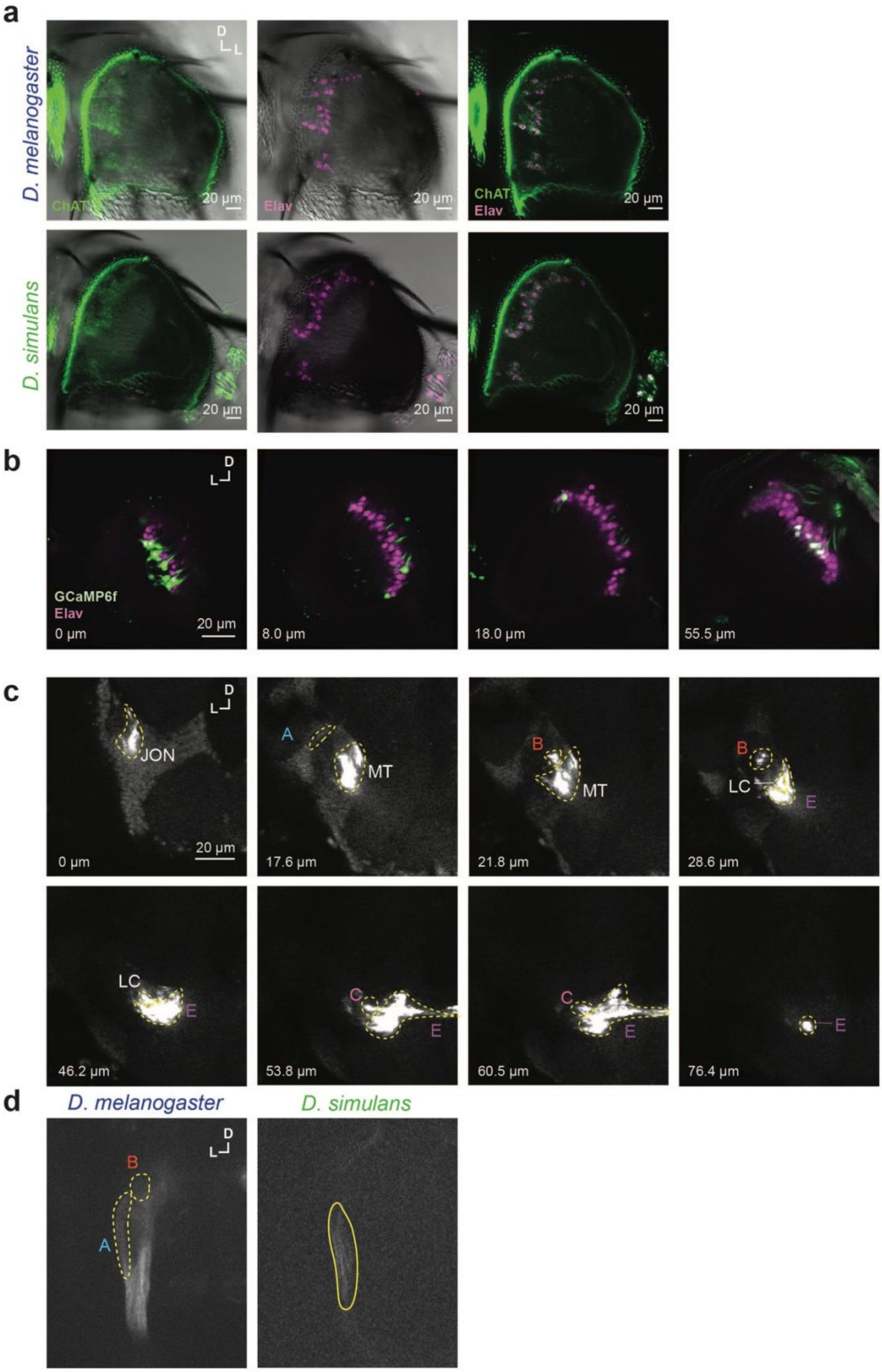

a
D. melanogaster
D. simulans
D
L
ChAT
Elav
20 μm
b
GCaMP6f
Elav
0 μm
8.0 μm
18.0 μm
55.5 μm
c
JON
MT
LC
A
B
C
E
0 μm
17.6 μm
21.8 μm
28.6 μm
46.2 μm
53.8 μm
60.5 μm
76.4 μm
d
D. melanogaster
D. simulans

Supplementary Figure S2. JO neurons labeled by anti-ChAT antibodies and *nanchung-GAL4* in *D. simulans*. (**a**) Somata of JO neurons (magenta) in both species labeled with anti-ChAT antibodies (green). Frontal confocal sections of JO within the second antennal segment are shown. Left, ChAT signals (green) and confocal transmission images (gray). Middle, elav signals (magenta) and confocal transmission images (gray) of the sections. Right, ChAT (green) and elav (magenta) signals are overlaid. D; Dorsal, L; Lateral. (**b**) GCaMP6f-labeled JO neurons (green) driven by *nanchung-GAL4* in *D. simulans*. Frontal confocal sections of immuno-labeled cell bodies of JO neurons in the second antennal segment are shown. Magenta signals represent nuclei of JO neurons labeled by anti-Elav antibody. D; Dorsal, L; Lateral. The depth from the most frontal panel was annotated at the bottom left of each panel. (**c**) Frontal confocal sections of the AMMC along the trajectory of labeled JO neuron axons in a *D. simulans* female brain. *nanchung-GAL4* was used as a driver to express a GCaMP6f marker. GCaMP6f signals were enhanced by anti-GFP antibodies. The fluorescent signals surrounded by dotted lines, which indicate the neurites projecting to the AMMC, are determined to be the axons of JO neurons (see Methods). D; Dorsal, L; Lateral. JON; JO neurons, MT; Main trunk, LC; Lateral core region, A; zone A, B; zone B, C; zone C, E; zone E. The depth from the most frontal panel was annotated at the left bottom of each panel. **(d)** Ventral view of GCaMP signals in JO neuron axons of *D. melanogaster* and *D. simulans*, detected with the calcium imaging setup. Dotted lines represent specific zones innervated by corresponding JO neurons in *D. melanogaster*. A; zone A, B; zone B (Left). A solid yellow line shows the ROI set at the axon bundle of labeled JO neurons in *D. simulans*, where the fluorescent increase is observable during sound stimulus (Right). D; Dorsal, L; Lateral.

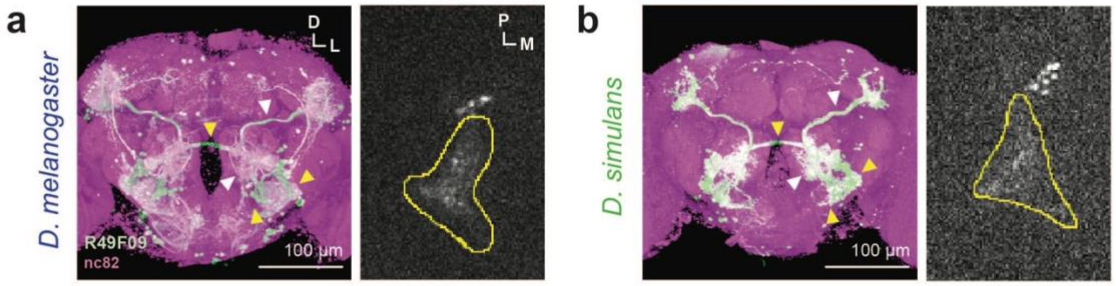

<u>Supplementary Figure S3.</u> Homologous neurons labeled by *R49F09-GAL4* in *D. melanogaster* and *D. simulans*. Labeling pattern (Left) and ROIs for calcium imaging (Right) of *R49F09-GAL4* females in *D. melanogaster* (**a**) and *D. simulans* (**b**) brains. For left panels, arrowheads indicate AMMC-B1 (yellow) and non-AMMC-B1 neurons (white). For right panels, the ROIs for the calcium imaging were set at the fluorescent region in the AMMC where AMMC-B1 neurons innervate but non-AMMC neurons do not (yellow line). D; Dorsal, L; Lateral, P; Posterior, M; Medial.

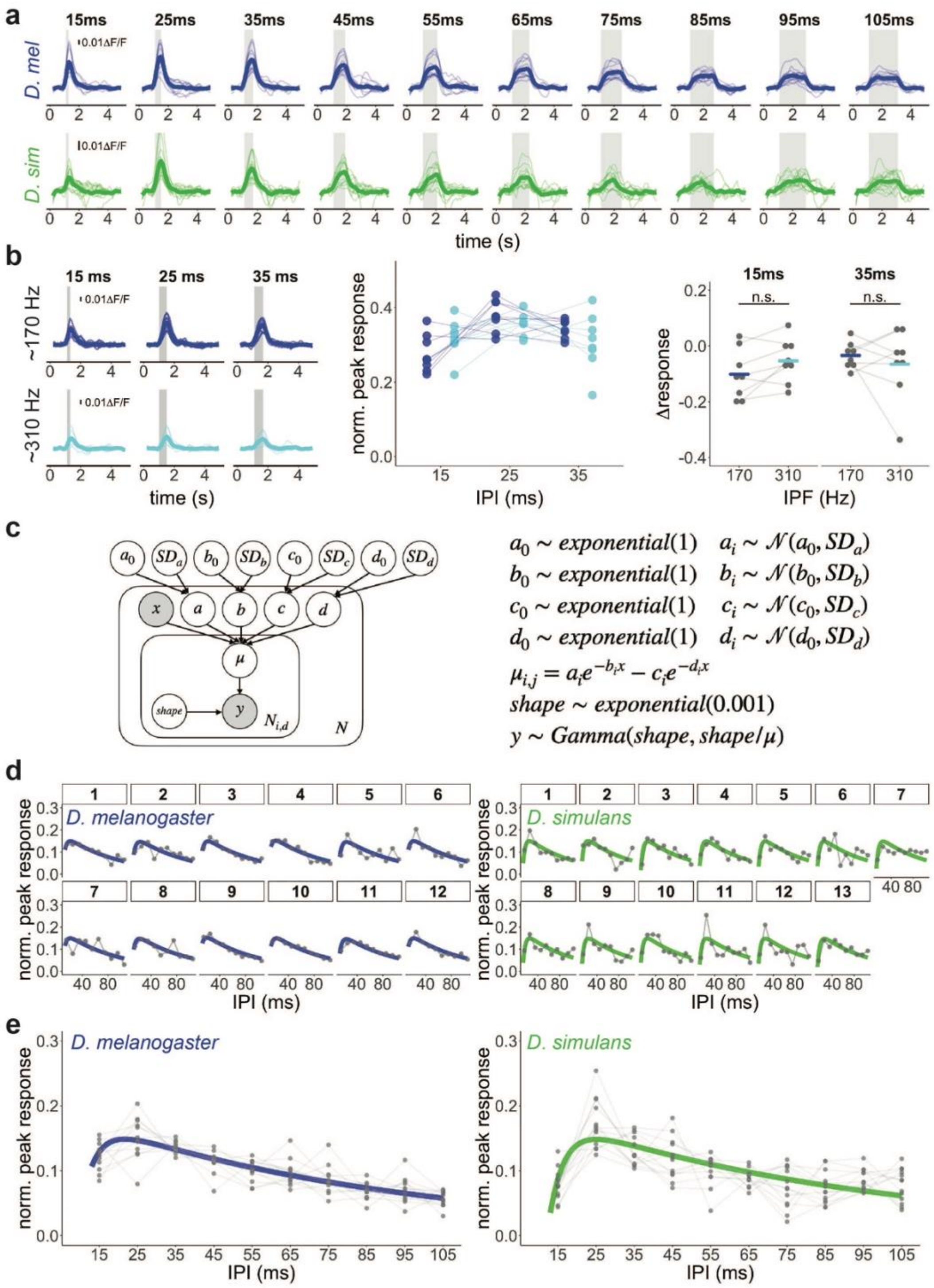

Supplementary Figure S4. IPI response properties of AMMC-B1 neurons. (**a**) Responses to artificial pulse songs with different IPIs in *D. melanogaster* (blue) and *D. simulans* (green). The sound stimulus was comprised of 20 pulses for each IPI song (shaded gray area). Thin and bold lines show time traces of the response in each individual and the average of all individuals, respectively. (**b**) IPF effect on the

IPI response property of AMMC-B1 neurons in *D. melanogaster*. Left: Responses to artificial pulse songs with two different IPFs (~170 Hz and ~310 Hz). The sound stimulus was comprised of 20 pulses for each IPI song (shaded gray area). Thin and bold lines show time traces of the response in each individual and the average of all individuals, respectively. Middle: Normalized peak responses of AMMC-B1 neurons to pulse songs with IPFs of ~170 Hz (blue) or ~310 Hz (cyan). Dots show normalized peak responses in each individual, where dots of the same individuals are connected with lines. Right: Differences from normalized peak responses to pulse song with 25 ms IPI (i.e., Δresponses) carrying an IPF of ~170 Hz (blue) or ~310 Hz (cyan). n.s.: $P > 0.05$; pairwise t-test with Bonferroni correction. Crossbars and dots show the median and individual data, respectively. Data obtained from the same individual were connected with a line. (**c**) Bayesian hierarchical model for fitting. gray circles represent input values and white circles denote estimated values. Parameters of each individual and the entire group were estimated. (**d**)-(**e**) Fitting curves of normalized peak response of each individual (**d**) and entire groups (**e**). Solid smooth lines represent the fitted curve. Dots show normalized peak responses in each individual. Data obtained from the same individuals are connected with lines.

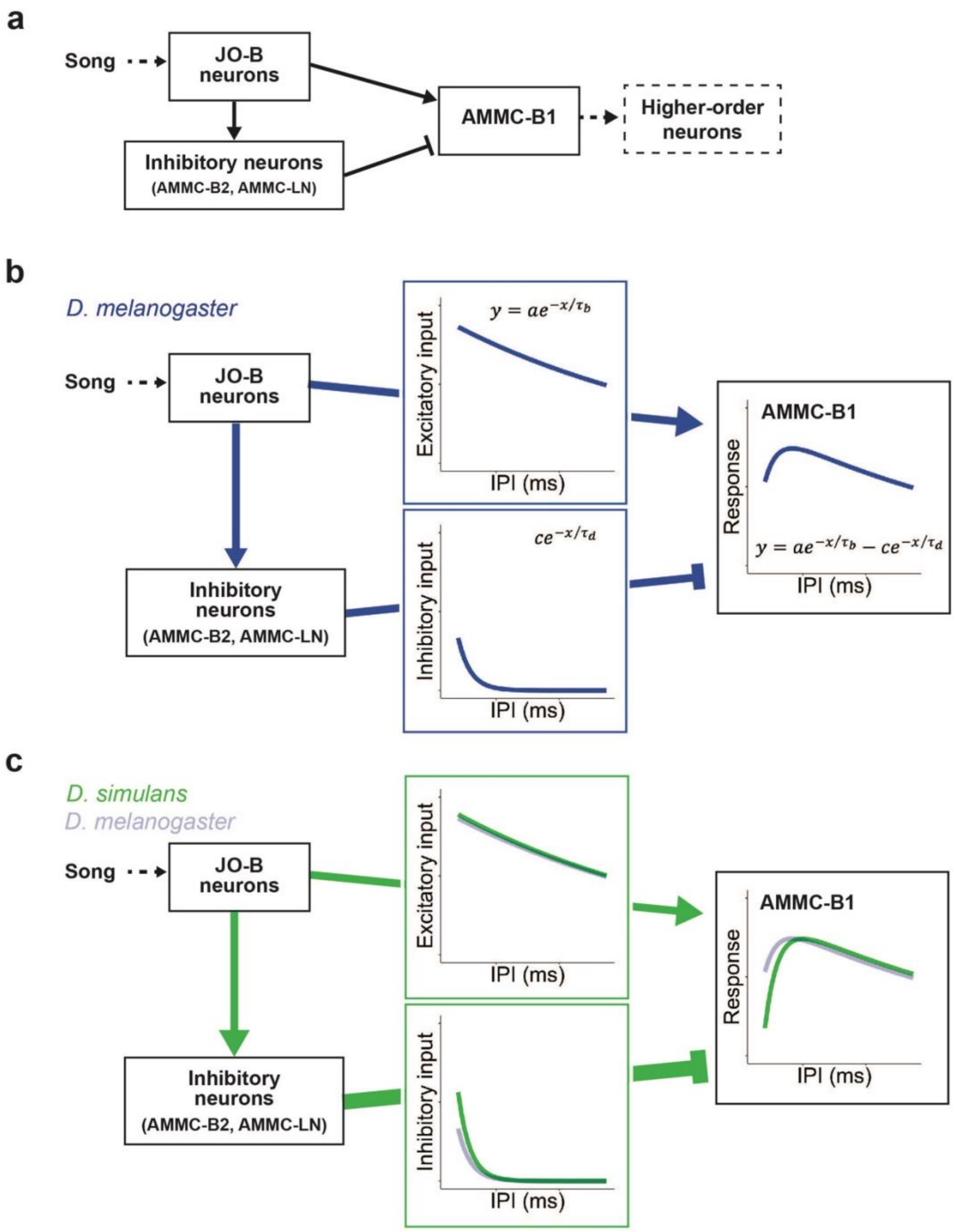

Supplementary Figure S5. A feed-forward auditory pathway composed of excitatory and inhibitory inputs to generate the interspecific difference in IPI response properties of AMMC-B1 neurons. (**a**) A model of the feed-forward auditory pathway in *D. melanogaster* inferred in a previous study[11]. JO-B neurons and two inhibitory neurons, AMMC-LN and AMMC-B2, modulate the response pattern of AMMC-B1 neurons. (**b**) IPI response properties of AMMC-B1 and excitatory and inhibitory input

properties from JO-B neurons and AMMC-B2/LN neurons, respectively, in *D. melanogaster*. The fitting curve of the neural response of AMMC-B1 and excitatory/inhibitory inputs are drawn based on the parameters estimated by the hierarchical Bayesian model in *D. melanogaster* (Fig. 4e). JO-B neurons and two inhibitory neurons, AMMC-B2 and AMMC-LN, send stronger inputs to AMMC-B1 at pulse songs with short IPIs. Both excitatory and inhibitory inputs shape the significant attenuation of the response of AMMC-B1 neurons to songs with a 15-ms IPI ($p = 0.01$). (**c**) Interspecific difference of IPI response properties of AMMC-B1 neurons shaped by excitatory and inhibitory inputs from JO-B neurons and AMMC-B2/LN neurons. The fitting curve of the neural response of AMMC-B1 and excitatory/inhibitory inputs are drawn based on parameters estimated by the hierarchical Bayesian model in *D. simulans* (green) and *D. melanogaster* (light blue) (see Table 1).

Supplementary Tables

Figure	Time window	Explanatory variable	HR	5% CI	95% CI	p value
Figure 1	0-7 min	IPI (35-15ms)	0.19	0.09	0.39	5.04E-06
		IPI (35-55ms)	0.73	0.44	1.21	0.217
		IPI (35-75ms)	0.87	0.52	1.46	0.598
		IPI (35-95ms)	0.55	0.32	0.95	0.033
		melanogaster-simulans	0.43	0.24	0.77	0.005
		(35-15)*(mel-sim)	1.44	0.45	4.65	0.538
		(35-55)*(mel-sim)	2.30	1.02	5.15	0.043
		(35-75)*(mel-sim)	1.01	0.42	2.41	0.979
		(35-95)*(mel-sim)	0.93	0.36	2.43	0.890
	7-30 min	IPI (35-15ms)	0.51	0.24	1.08	0.078
		IPI (35-55ms)	0.85	0.37	1.95	0.696
		IPI (35-75ms)	0.70	0.29	1.70	0.432
		IPI (35-95ms)	0.72	0.32	1.63	0.427
		melanogaster-simulans	0.68	0.32	1.47	0.328
		(35-15)*(mel-sim)	0.74	0.26	2.12	0.574
		(35-55)*(mel-sim)	0.98	0.31	3.05	0.971
		(35-75)*(mel-sim)	0.64	0.20	2.09	0.459
		(35-95)*(mel-sim)	0.88	0.30	2.58	0.809
Supplementary Figure 1b	0-7 min	IPI (35-15ms)	0.16	0.07	0.35	8.76E-06
		IPI (35-55ms)	0.73	0.43	1.22	0.229
		melanogaster-simulans	1.05	0.62	1.76	0.864
		(35-15)*(mel-sim)	1.51	0.50	4.51	0.463
		(35-55)*(mel-sim)	1.20	0.56	2.55	0.643
	7-30 min	IPI (35-15ms)	0.38	0.20	0.72	0.003
		IPI (35-55ms)	0.43	0.21	0.90	0.025
		melanogaster-simulans	0.19	0.07	0.49	0.001
		(35-15)*(mel-sim)	2.37	0.74	7.52	0.144
		(35-55)*(mel-sim)	5.09	1.46	17.80	0.011

<u>Table S1.</u>

Hazard ratio of copulation assay in females.

(35-X)*(mel-sim) represents the interaction between IPI (35 ms and X ms) and species (*D. melanogaster* and *D. simulans*). HR; Hazard ratio. CI; Confidence interval.

Figure	Experiment	Statistical method	Group	p value	Statistics	Value
2A	Cell number	ART ANOVA	-	2.290E-04	Df.res	1
					Df.res	10
					F value	3.13E+01
3B	NBLAST	ART ANOVA	-	0.821	Df.res	1
					Df.res	10
					F value	5.42E-02
3D, E	Interspecific comparison of frequency characteristics of AMMC-B1	GLM (distribution = Gamma , link function = log)	Intercept	5.350E-04	Estimate	-0.65
					Standard error	0.17
					t value	-3.74
			Frequency	0.029	Estimate	-0.553
					Standard error	0.24
					t value	-2.26
			Species	8.540E-15	Estimate	-9.18E-03
					Standard error	8.01E-04
					t value	-11.46
			Species*Frequency	5.587E-03	Estimate	3.30E-03
					Standard error	1.13E-03
					t value	2.91
4D	⊿response	t-test adjusted by Bonferroni	IPI 15 and 25 ms	0.010	t value	-3.73
					Df.res	22.92
					p value before adjusted	1.12E-03
			IPI 35 and 25 ms	1.000	t value	-1.60
					Df.res	22.6

		p value before adjusted	0.12
		t value	-0.83
		Df.res	20.57
IPI 45 and 25 ms	1.000	p value before adjusted	0.42
		t value	-1.09
		Df.res	22.77
IPI 55 and 25 ms	1.000	p value before adjusted	0.29
		t value	-1.65
		Df.res	18.04
IPI 65 and 25 ms	1.000	p value before adjusted	0.12
		t value	-1.67
		Df.res	21.64
IPI 75 and 25 ms	0.993	p value before adjusted	0.11
		t value	-1.01
		Df.res	22.22
IPI 85 and 25 ms	1.000	p value before adjusted	0.32
		t value	-0.35
		Df.res	21.49
IPI 95 and 25 ms	1.000	p value before adjusted	0.73
		t value	-0.049
IPI 105 and 25 ms	1.000	Df.res	18.89

					p value before adjusted	0.96
4F	Comparison of estimated parameters in model fitting	Exact Wilcoxon rank sum test	a	3.846E-07	W	0
			tau_b	1.538E-06		154.00
			c	3.846E-07		0
			tau_d	0.011		32.00
			peak	3.846E-07		0
S4B Right	Δresponse	pairwise t-test adjusted by Bonferroni	IPI 15 and 25 ms	0.113	t value	-2.28
					Df.res	7.00
					p value before adjusted	0.056
			IPI 35 and 25 ms	0.990	t value	0.72
					Df.res	7.00
					p value before adjusted	0.49

Table S2.

Statistical results.

Detailed statistical results in each figure are listed. Df.res; Degrees of freedom of residuals.

Target	Species	Driver strain	Effector strain	Genotype	Figure
-	*D. melanogaster*	-	-	*+; +; +*<br>*(Canton-S originated from Hotta lab)*	1B-D, 2A, D
-	*D. simulans*	-	-	*w; +; UAS-GCaMP6f*<br>*(2034 Sim w4 pBac(GCamp6F)5)*	1, 2B
-	*D. simulans*	-	-	*+; +; +*<br>*(Flybase ID: FBst0201373)*	2A, D
-	*D. melanogaster*	-	-	*w; UAS-GCaMP6f; +*	2B
JON	*D. melanogaster*	*F-GAL4*	*UAS-GCaMP6f*	*w; F-GAL4, UAS-GCaMP6f; +*	2C
JON	*D. simulans*	*nanchung-GAL4*	*UAS-GCaMP6f*	*w/yw; +; UAS-GCaMP6f(2034)/ nanchung-GAL4 (TG-S15)*	2B, F, Sup2
AMMC-B1	*D. melanogaster*	*R49F09-GAL4*	*UAS-GCaMP6f*	*w; UAS-GCaMP6f/+; R49F09-GAL4/+*	3, 4, Sup 1B-C Sup3, Sup4
AMMC-B1	*D. simulans*	*R49F09-GAL4*	*UAS-GCaMP6f*	*w/yw; R49F09-GAL4 (2176)/+; UAS-GCaMP6f (2034)/+*	3, 4, Sup 1B-C, Sup3, Sup4

Table S3.

Genotypes of flies used in this study.

Figure	Experiment	Species	Group	Sample size
1	Female copulation assay	*D. melanogaster*	15ms-IPI	44
			35ms-IPI	46
			55ms-IPI	43
			75ms-IPI	38
			95ms-IPI	39
		D. simulans	15ms-IPI	40
			35ms-IPI	40
			55ms-IPI	40
			75ms-IPI	40
			95ms-IPI	40
2A	The cell number of JO neurons	*D. melanogaster*	-	6
		D. simulans	-	6
2F	Calcium imaging of JO neurons	*D. simulans*	-	5
3C	NBLAST	*D. melanogaster*	-	5
		D. simulans	-	7
3E, F	Calcium imaging of AMMC-B1 (Pure tone)	*D. melanogaster*	-	8
		D. simulans	-	8
4, Sup4A, C-E	Calcium imaging of AMMC-B1 (IPI)	*D. melanogaster*	-	12
		D. simulans	-	13
Sup1B-C	Female copulation assay	*D. melanogaster*	15ms-IPI	45
			35ms-IPI	47
			55ms-IPI	47
		D. simulans	15ms-IPI	44
			35ms-IPI	40
			55ms-IPI	40
Sup4B	Calcium imaging of AMMC-B1(IPF)	*D. melanogaster*	-	8

<u>Table S4.</u>

Sample sizes for each experiment.

Parameter	Starting value
a0	0.1
b0	0.01
c0	0.1
d0	0.1
a	0.1
b	0.01
c	0.1
d	0.1
SD_a	0.005
SD_b	0.005
SD_c	0.005
SD_d	0.005
shape	50

Table S5.

Starting values of the parameters used for MCMC fitting (See Supplementary Fig. S4).

Figure	Time window	Explanatory variable	chi-square	Df	p value
1	0-30 min	IPI	0.15	4	5.5E-03
		Species	5.88E-04	1	0.98
		IPI*Species	9.72	4	4.5E-02
		GLOBAL	0.17	9	4.6E-02
	0-7 min	IPI	3.53	4	0.47
		Species	2.19	1	0.14
		IPI*Species	3.96	4	0.41
		GLOBAL	7.83	9	0.55
	7-30 min	IPI	4.09	4	0.39
		Species	0.39	1	0.53
		IPI*Species	4.61	4	0.33
		GLOBAL	7.26	9	0.61
Sup1B-C	0-30 min	IPI	10.92	2	0.0042
		Species	12.55	1	0.0004
		IPI*Species	1.72	2	0.4235
		GLOBAL	27.02	5	5.6e-05
	0-7 min	IPI	0.556	2	0.76
		Species	2.398	1	0.12
		IPI*Species	0.936	2	0.63
		GLOBAL	4.758	5	0.45
	7-30 min	IPI	6.718	2	0.035
		Species	0.223	1	0.636
		IPI*Species	2.927	2	0.231
		GLOBAL	7.133	5	0.211

Table S6.

Proportional assumption of cox hazard proportional model.

When p value of GLOBAL is less than 0.05, hazard proportionality is rejected. Df; Degree of freedom.
